# Expression of a Recombinant Anti-HIV and Anti-Tumor Protein, MAP30, in *Nicotiana tobacum* Hairy Roots: A pH-Stable and Thermophilic Antimicrobial Protein

**DOI:** 10.1371/journal.pone.0159653

**Published:** 2016-07-26

**Authors:** Ali Moghadam, Ali Niazi, Alireza Afsharifar, Seyed Mohsen Taghavi

**Affiliations:** 1 Institute of Biotechnology, Shiraz University, Shiraz, Iran; 2 Plant Virology Research Center, College of Agriculture, Shiraz University, Shiraz, Iran; 3 Department of Plant Protection, College of Agriculture, Shiraz University, Shiraz, Iran; USDA-ARS, UNITED STATES

## Abstract

In contrast to conventional antibiotics, which microorganisms can readily evade, it is nearly impossible for a microbial strain that is sensitive to antimicrobial proteins to convert to a resistant strain. Therefore, antimicrobial proteins and peptides that are promising alternative candidates for the control of bacterial infections are under investigation. The MAP30 protein of *Momordica charantia* is a valuable type I ribosome-inactivating protein (RIP) with anti-HIV and anti-tumor activities. Whereas the antimicrobial activity of some type I RIPs has been confirmed, less attention has been paid to the antimicrobial activity of MAP30 produced in a stable, easily handled, and extremely cost-effective protein-expression system. rMAP30-KDEL was expressed in *Nicotiana tobacum* hairy roots, and its effect on different microorganisms was investigated. Analysis of the extracted total proteins of transgenic hairy roots showed that rMAP30-KDEL was expressed effectively and that this protein exhibited significant antibacterial activity in a dose-dependent manner. rMAP30-KDEL also possessed thermal and pH stability. Bioinformatic analysis of MAP30 and other RIPs regarding their conserved motifs, amino-acid contents, charge, aliphatic index, GRAVY value, and secondary structures demonstrated that these factors accounted for their thermophilicity. Therefore, RIPs such as MAP30 and its derived peptides might have promising applications as food preservatives, and their analysis might provide useful insights into designing clinically applicable antibiotic agents.

## Introduction

The increase in microbial resistance to conventional antibiotics and the need for new antibiotics has encouraged the development of antimicrobial proteins and peptides [1–5]. The great potential of natural antimicrobial proteins and peptides derived from medicinal plants to play a role in fighting infections in humans and pathogens in plants has been documented [6–8]. The advantage of antimicrobial proteins and peptides over conventional antibiotics, such as penicillin, is that a microbial strain sensitive might not mutate into a resistant strain [1].

However, it is generally difficult and very costly to purify a specific protein from natural host cells [9]. Therefore, expressing the antimicrobial genes in a suitable host is an effective practical solution to these problems [10–12]. In this regard, prokaryotic and eukaryotic recombinant protein-expression systems (RPESs) such as bacterial, fungal, insect cell-, mammalian cell-, and plant-based systems, have developed [1,3,13]. Unlike eukaryotic cells, prokaryotic cells have certain limitations, such as the inability to perform appropriate posttranslational modifications (PTMs) of specific amino acids [14], inefficient protein cleaving and folding [15], and the unsuitable formation of disulfide bonds in cysteine-rich peptides [16], and therefore produce recombinant proteins that are often misfolded and form inactive inclusion bodies [13–14]. Thus, plant-based systems have been considered as valuable platforms for producing eukaryotic recombinant proteins, even those that are beneficial for human health [14,17]. Studies have demonstrated that molecular farming in plants has many practical, economical and safety advantages over conventional systems because of its well-documented potential for the adaptable and extremely cost-effective production of bioactive and efficacious proteins on a large scale [13,18–19]. Therefore, plant-based RPESs (PBRPESs) are gaining increased acceptance [11,13,17]. Moreover, the level of plant PTMs resulting in the production of proteins that are toxic to animals in these systems [3,12] is similar to that of mammalian cells, with slight differences in the glycan residue-associated metals that do not appear to affect the particular immunogenicity of the target product [3]. Finally, PBRPESs are safer than traditional production systems because of their lack of contamination with extraneous animal viral or bacterial materials or mammalian pathogens and because their products are more authentic [9–10,20].

Nevertheless, extracting and purifying intricate biopharmaceutical proteins from whole plants are time-consuming and costly processes [21]. As a result, in vitro plant-cell cultures and particularly, hairy roots (HRs) are used as alternatives to whole plants for the production of recombinant proteins [21–23]. As PBRPES, HRs secrete properly folded functionally active recombinant proteins into the culture medium or retain them within their cells [21]. HRs are neoplastic tissues that result from the *rol* loci of *Agrobacterium rhizogenes* being transformed into the host-cell genome [21,24]. HR cultures are maintained in a simple medium containing a mixture of sucrose and salts that is free of hormones and of any products of animal origin [25]. The advantages of rapidly growing HRs over other plant-based systems, such as suspended cells, include efficient productivity, long-term genotype and phenotype stability, rapid biomass production on a commercial level, differentiated organ-specific cultures of clonal origin [19,25], time savings, and constancy in the expression of the target gene over a long period [26]. An inexpensive and simple expression system for the large-scale production of safe recombinant proteins is greatly needed [3]. Because the first recombinant protein, murine IgG1, was successfully produced in HRs [27], other recombinant proteins, such as enzymes [28], human secreted alkaline phosphatase [21], growth factors [29], and reporter proteins [30], have been expressed by HRs at levels of three- to five-fold higher than those of their parental transgenic plants [28]. In addition, subcellular targeting plays a significant role in determining the yield of recombinant proteins because the compartment in which a recombinant protein accumulates highly affects the interrelated processes of folding, assembly, and PTM [26]. Fusing a C-terminal KDEL (Lys-Asp-Glu-Leu) sequence to the target protein to retain it in the endoplasmic reticulum (ER) has been shown to increase its stability [31]. The yields of recombinant proteins retained in the ER are generally two to ten times greater than those of secreted recombinant proteins [3]. The ER provides an oxidizing environment with an abundance of molecular chaperones and few proteases [26,28]. These features are the most important factors affecting protein stability, folding, and assembly [26,32]. The levels of accumulation of KDEL-tagged proteins are generally 2 to 10-fold greater than those of proteins that lack this signal [32]. Considering all of the aspects mentioned above, the cost of protein production in PBRPESs, particularly in HRs, could be approximately 1000 times less than those utilizing traditional expression systems [3,20].

**Fig 1:**
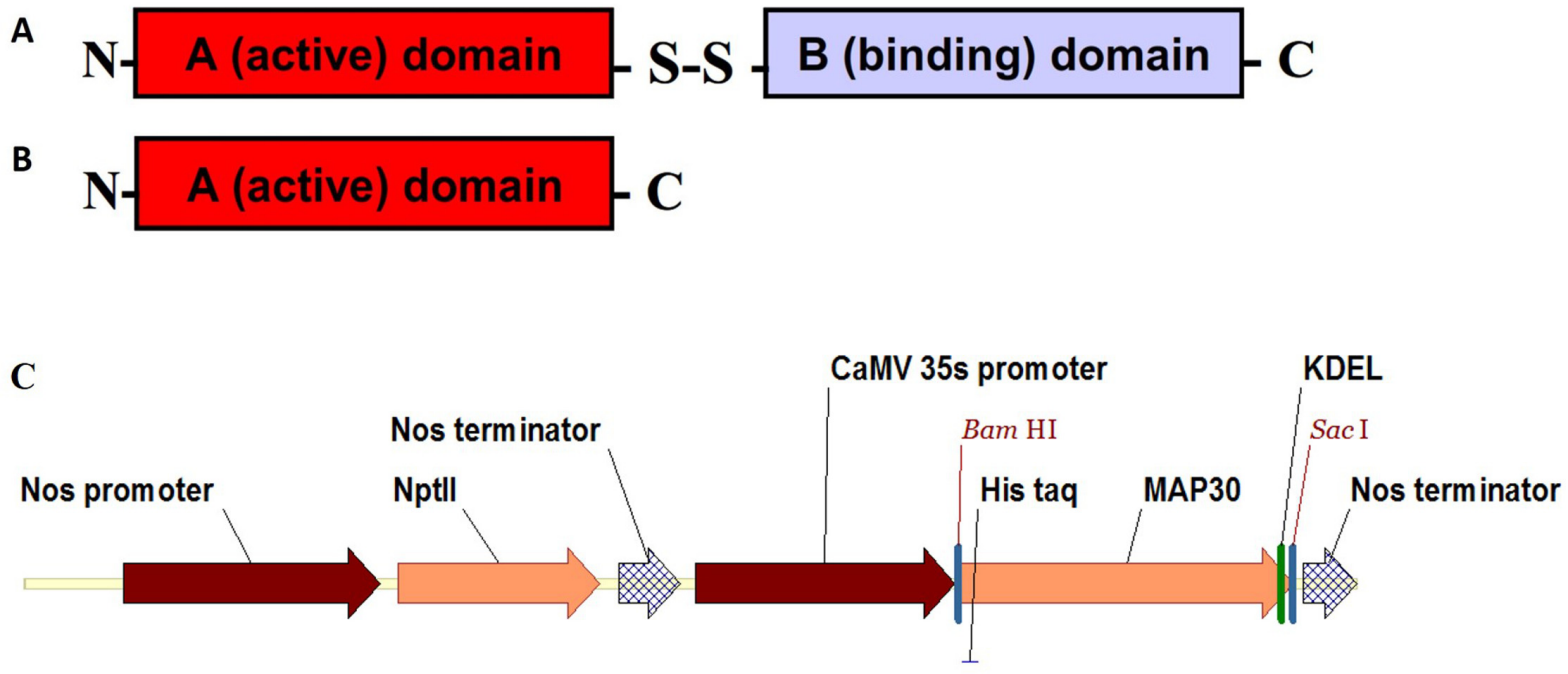
Two types of RIPs and the T-DNA regions of the binary plasmids used for *A*. *rhizogenes* transformation. Type II RIPs are comprised of two dissimilar chains including an A (Active domain) chain and a B (Binding domain) chain (A), whereas type I RIPs are single-chain proteins that consist of an A (Active domain) chain (B). Plasmid pBI121 contained the *npt*II (kanamycin-resistance) gene under the control of the nopaline synthase (NOS) gene promoter and a codon-optimized *MAP30* CDS under the control of the CaMV 35S promoter and with a nos terminator. The 6×His tag and the ER-retention signal KDEL were fused at the N- and C-terminus in-frame with the *MAP30* CDS, respectively (C).

Groups of antimicrobial proteins with established activity against fungi and bacteria play a crucial role in the plant-defense system protecting a plant host against pathogen attacks [33]. These proteins include ribosome-inactivating proteins (RIPs), defensins, pathogenesis-related proteins, lipid-transferring proteins, cyclotides, lectins, digestive-enzyme inhibitors, and chitin-binding proteins [6]. Among these proteins, RIPs are broadly dispersed in nature but are found mainly in plants, fungi, and bacteria [34]. Type II RIPs are composed of two dissimilar chains, including an A (Active) chain and a B (Binding) chain (Fig 1A and 1B) [34–35]. The B chain is a lectin that binds to the galactose residues on cellular membranes, leading to the entry of the RIP molecule into cells, where the A chain enzymatically destroys ribosomes and eventually kills the cells [33]. However, single-chain type I RIPs hardly penetrate cells due to their lack of a lectin domain and much lower cytotoxicity [33–34].

In addition to rRNA N-glycosidase activity [36], some RIPs exhibit various antimicrobial activities in vitro, such as antifungal, antibacterial, and antiviral activities against both animal and human viruses [34]. A type I RIP called MAP30 was previously found to have anti-HIV (human immunodeficiency virus) and anti-tumor activities [37–39]. MAP30 was purified from the seeds and fruits of *Momordica charantia* [36]. This tropical plant has been shown to have therapeutic properties, such as antidiabetic, antineoplastic, and antioxidant activities [8,35–37]. Extracts of this plant also exhibited antimicrobial activities against fungi and against Gram-negative and Gram-positive bacteriaand parasitic organisms [35].

Recently, there has been renewed interest in MAP30 due to reports of its potent antitumor and antimicrobial activities [37,40–41], although the molecular mechanisms underlying these activities are not fully elucidated [7–8,40–41]. MAP30 also selectively attacks HIV-infected cells and tumor-transformed cells without harming healthy cells [35,37]. In addition, when delivered via an essential in vivo drug delivery system, MAP30 had a reduced level of anti-HIV activity but a prolonged in vivo half-life [42]. Due to the trivial MAP30 content of *M*. *charantia*, recombinant MAP30-KDEL (rMAP30-KDEL) was expressed in *Nicotiana tobacum* hairy roots for the first time and its antimicrobial activity, thermal and pH stabilities, and physical properties were investigated.

## Materials and Methods

### Growth conditions of the plant source

Seeds of *N*. *tobacco* L. cv. Turkish were obtained from the Plant Virology Research Center, College of Agriculture, Shiraz University, Shiraz-Iran. The seeds were sterilized by soaking them in 70% (v/v) ethanol for 30 sec, then soaking them in a 2% hypochlorite sodium for 10 min, and finally rinsing those five times using sterile distilled water. The sterilized seeds were grown on solid Murashige and Skoog (MS) medium [43] for 2 weeks at 25°C with a 16/8 h light/dark photoperiod.

### Construction of the expression vector for MAP30-KDEL

The coding region (CDS) of *MAP30*, which contains 861 bp, was commercially synthesized (Biomatik, Canada). Its codon optimization was based on the codon-usage bias of the host *N*. *tobacco*. The *MAP30* CDS was inserted into the pBI121 expression vector through the *Bam*HI and the *Sac*I sites and a recombinant pBI121-MAP30-expression vector was designed. In this vector, which contained ampicillin- and kanamycin-resistance genes, the expression of the *MAP30* CDS was under the control of the CaMV 35S promoter and the nopaline synthase (NOS) terminator. In addition, a 6×His tag and the ER-retention signal KDEL were fused at the N- and C-terminus, respectively, in-frame with the *MAP30* CDS (Fig 1C).

### Construction of the pBI121-MAP30 expression vector

The synthetic pBI121-MAP30 expression vector was diluted 10 times, and 2 μL of this sample was used to transform *E*. *coli* strain DH5α using the electroporation method. These bacteria were then dispersed on Luria-Bertani (LB) agar supplemented with 50 mg/L of kanamycin and were incubated at 37°C overnight. Single colonies were selected and were cultured in liquid LB medium supplemented with 50 mg/L of kanamycin with agitation at 37°C overnight. Transformation of the colonies was confirmed using a specific PCR assay and by digestion of the extracted plasmid.

### Transformation of *A*. *rhizogenes*

The plasmid was extracted from transformed *E*. *coli* using a Plasmid Miniprep Kit (Fermentas). The recombinant plasmid was diluted 10 times, and 10 μL of this sample was used to transform 100 μL of *A*. *rhizogenes* strain ATCC AR15834 (at OD_600 nm_ = 1) using the freeze-thaw method. One milliliter of liquid LB was added, and the cells were incubated at 28°C in the dark for 2 h. Then, the transformed bacteria were dispersed on LB agar containing kanamycin and rifampicin (100 mg/L) and were incubated at 28°C in the dark for 48 h. Colonies were confirmed to be recombinant using a specific PCR assay and by digestion of the extracted plasmid.

### Regeneration, growth and establishment of hairy roots

Leaf explants of 1–2 cm were inoculated for 5 min with an *A*. *rhizogenes* culture that had been grown overnight (to OD_600 nm_ = 0.5), and then these materials were co-cultured on solid MS medium at 25°C in the dark. After three days, the explants were transferred to fresh MS medium supplemented with 400 mg/L of cefotaxime and 150 mg/L of kanamycin and were maintained at 25°C under a 16/8 h light/dark photoperiod for two weeks. The hairy roots that formed at the incision sites of the leaf fragments were subsequently transferred at two-weeks intervals to fresh MS agar containing cefotaxime and kanamycin at the concentrations noted above and were incubated at 25°C in the dark.

### Development of the culture conditions for hairy root maintenance

After sub-culturing replicates of the transformed hairy root clones several times, DNA and RNA were extracted and then the hairy roots confirmed to be transgenic were transferred to a 250-ml Erlenmeyer flask containing liquid MS medium without antibiotics and were grown at 28°C in the dark with mild shaking for one or two months for protein extraction; the medium was refreshed weekly.

### DNA and RNA extraction and cDNA synthesis

Genomic DNA was extracted using the modified CTAB method [44]. Total RNA was extracted using an RNX-Plus reagent kit (Cinnagen, Tehran, Iran) according to the manufacturer’s instructions. Then, the quantity and concentration of the RNA and DNA were measured using a Nanodrop device (Thermo Fisher Scientific, USA). The integrity and quantity of RNA were evaluated by visual observation of the 28S and 18S rRNA bands on a 1% agarose gel. Then, cDNAs were synthesized using a first-strand cDNA synthesis kit (Fermentas, Germany) according the manufacturer’s instructions. DNA-free total RNA (1 μg) was reverse transcribed using oligo-dT primers (Fermentas). The cDNA samples were stored at -20°C until use.

### Screening transgenic hairy roots

Primers specific for *rolB*, *MAP30*, and *virG* were designed using Allele ID 7 and Vector NTI 11 software (Table 1). The resulting PCR primers were used to amplify the *MAP30* cDNA and DNA that was extracted from hairy root samples. Then, primers specific for the amplification of *rolB* in the transgenic hairy roots and primers specific for the amplification of *virG* were used to confirm that the *A*. *rhizogenes* infection had been eliminated.

**Table 1:** Sequences of the primers used for PCR-based characterization of transgenic hairy roots and the resulting product sizes. The primers were designed using Vector NTI 11 and Allele ID 7 software.

Primer (bp)	Sequence	Ta (C°)	Product length (bp)
*MAP30-*F	ATGGCACCACAAAAGGAGAAC	58	861
*MAP30-*R	AACCTGAAACCTTTCTCCTGTAG		
*rolB-*F	AAGTGCTGAAGGAACAATC	54	194
*rolB-*R	CAAGTGAATGAACAAGGAAC		
*virG-*F	CCTTGGGCGTCGTCATAC	55	529
*virG-*R	TCGTCCTCGGTCGTTTCC		

*T*_a_, temperature annealing; *F*, Forward; *R*, Reverse

### Extraction of total proteins

The total proteins of 6 transgenic hairy root clones (5 g) were extracted using phosphate buffer (100 mM, pH 7). First, the hairy root clones were ground under liquid nitrogen, and the powder was suspended in 1:1 phosphate buffer w/v. Then, the supernatant was prepared by centrifugation at 4000 ×g for 10 min at 4°C. The amount of extracted total protein was determined using the Bradford method [45], and the proteins were stored at -20°C prior to use.

### Protein purification under native conditions

The recombinant protein was purified using a Ni-NTA spin column (cat. No. 31014, Qiagen). First, the Ni-NTA spin column was equilibrated by loading 600 μL of lysis buffer (50 mM NaH_2_PO_4_, 300 mM NaCl, 10 mM imidazole, pH 8.0) and centrifuging it for 2 min at 890 ×g. Next, up to 600 μL of concentrated root extract containing 6×His-tagged MAP30 was loaded onto the column and it was centrifuged for 5 min at 270 ×g, and the flow-through was collected. Then, the column was washed twice with 600 μL of wash buffer (50 mM NaH_2_PO_4_, 300 mM NaCl, 20 mM imidazole, pH 8.0) by centrifugation for 2 min at 890 ×g. Finally, the protein was eluted twice using 300 μL of elution buffer (50 mM NaH_2_PO_4_, 300 mM NaCl, 500 mM imidazole, pH 8.0) with centrifugation for 2 min at 890 ×g, and then the protein was aliquoted and was stored at 80°C.

### SDS-PAGE

The total proteins extracted from the transgenic and non-transgenic hairy root clones were separated by sodium dodecyl sulfate-polyacrylamide gel electrophoresis (SDS-PAGE) using a 12% polyacrylamide gel [46]. Following electrophoresis, the gel was stained using Coomassie brilliant blue.

### Microbial strains and antimicrobial-activity assays

Studies of the antimicrobial activity of rMAP30-KDEL were performed using Gram-positive bacterial strains, including *Streptococcus epidermidis* PTCC 1114 (ATCC 12228), *S*. *aureus* PTCC 1112 (ATCC 6538), and *Bacillus subtilis* PTCC 1023 (ATCC 6633), Gram-positive bacterial strains, including *Salmonella typhi* PTCC 1609, *E*. *coli* PTCC 1330 (ATCC 8739), *Pseudomonas aeruginosa* PTCC 1074 (ATCC 9027), *Xanthomonas citri*, *Ralstonia solanacearum*, and *P*. *syringae* pathovar syringae, and fungal strains, including *Leuconostoc mesenteroides* PTCC 1059 (ATCC 10830a), *Aspergillus niger* PTCC 5010 (ATCC 10864), and *Candida albicans* PTCC 5027 (ATCC 10231). The animal-specific bacterial and fungal strains were supplied by the Iranian Research Organization for Science and Technology (IROST), and the plant-specific bacterial species were supplied by the Department of Plant Protection, College of Agriculture, Shiraz, Iran.

The antimicrobial activity of the total protein extracted from the transgenic hairy roots was determined using the disc-diffusion method [47]. Briefly, following overnight cultivation of the bacterial and fungal species, 50 μL of a suspension containing 10^8^ CFU/mL of each species was inoculated into 50 mL of pre-warmed (45°C) nutrient agar (NA). After mixing, the medium was poured into sterile plates. Finally, four concentrations of the total protein samples (40, 80, 120, and 160 μg) were loaded on 6-mm sterile paper discs. The loaded discs were dried and were placed on the NA plates at 4°C for 2 h, and then the plates were incubated at 37°C for 16 h. Antibiotics (10 μg/disc) specific for Gram-negative and Gram-positive bacteria and fungi, which were gentamycin, ampicillin and ketoconazole, respectively, were used as positive controls. In addition, 160 μg of the total proteins extracted from non-transgenic hairy roots were used as a negative control.

### Thermal stability of rMAP30-KDEL

To study the temporal stability of rMAP30-KDEL, the total proteins extracted from transgenic and non-transgenic hairy roots were boiled for 1, 2, 3, 4, and 5 h, and the antibacterial activity of 80 μg of the boiled total proteins against *E*. *coli* was evaluated 16 h after cultivation at 37°C.

### Stability of rMAP30-KDEL at different pH values

To evaluate the stability of rMAP30-KDEL at different pH values, solutions containing the total proteins extracted from the transgenic hairy root clones were adjusted to pH 2, 3, 4, 5, 7, 8 or 9 by the dropwise addition of either 100 mM HCl or 100 mM NaOH and were stored overnight in a refrigerator. Then, the samples were allowed to equilibrate to room temperature and the level of antimicrobial activity against *E*. *coli* was determined at 1, 6, and 12 days of exposure.

### Effect of pH and temperature on the antibacterial activity of rMAP30-KDEL

To analyze the interaction between the effects of pH and temperature on the antibacterial activity of rMAP30-KDEL, after pH adjustment, the total proteins extracted from non-transgenic and transgenic hairy root clones were incubated at 80°C for 20 min, and then the antibacterial activity of 80 μg of the heat-treated total proteins against *E*. *coli* was evaluated after 16 h of cultivation.

### DNase activity of rMAP30-KDEL

To evaluate the topological-inactivation activity of rMAP30, 1 μg of plasmid DNA (pBI121) and genomic DNA was incubated with 40 μg of the total proteins extracted from transgenic and non-transgenic hairy roots in a 20-μL reaction volume (50 mM phosphate buffer, pH 7, 10 mM MgCl_2_ and 100 mM KCl) at 4, 25, and 37°C for 1 h. Equal amounts of the digested DNA samples were electrophoresed on 1% agarose gels and were visualized by ethidium bromide staining.

### Bioinformatic analysis

The amino-acid sequences of MAP30 (GenBank accession no. AAB35194) and other well-known RIPs from the following plant species were obtained from NCBI: the A chain of ricin from *Ricinus communis* (ABG65738), trichosanthin (AAO72728) and karasurin (P24478) from *Trichosanthes kirilowii*, GAP31 from *Suregada multiflora* (P33186), and curcin 1 from *Jatropha curcas* (ADN39429). The degree of identity of these sequences was assessed using different NCBI programs, such as BLAST-P and PSI-BLAST (http://BLAST.ncbi.nlm.nih.gov/BLAST.cgi), and vector NTI 11 software, and multiple-sequence alignment was performed using CLC Main Workbench 5 software (Qiagen). Then, the MEME (http://meme-suite.org/tools/meme) platform was used to identify patterns in the RIPs and the presence of any conserved motifs and domains; the presence of these patterns was screened by producing the multiple sequence alignments that were used to create the corresponding domain profile.

The ProtParam (http://web.expasy.org/protparam) was employed to determine various properties, such as the distribution of amino-acid residues, the theoretical pI, aliphatic index, charge, hydrogen bonding sites, and the grand average of hydropathicity (GRAVY) value. The amino-acid sequences of the RIPs were analyzed using several secondary-structure prediction tools in the SOPMA (https://npsa-prabi.ibcp.fr/cgi-bin/npsa_automat.pl?page=/NPSA/npsa_sopma.html) platform, which generated a consensus secondary structure.

### Statistical analysis

All the analyses were performed in triplicate, the mean values of the diameters of the inhibition zones were calculated, and the data sets were subjected to an analysis of variance (ANOVA) and Duncan’s multiple range test using MINITAB 16 (Minitab, Inc., Pennsylvania, USA) and SPSS 21 software. In all cases, a *P* value of ≤ 0.01 was considered significant.

## Results and Discussion

### Molecular and morphological characterization of the transgenic hairy root clones

**Fig 2:**
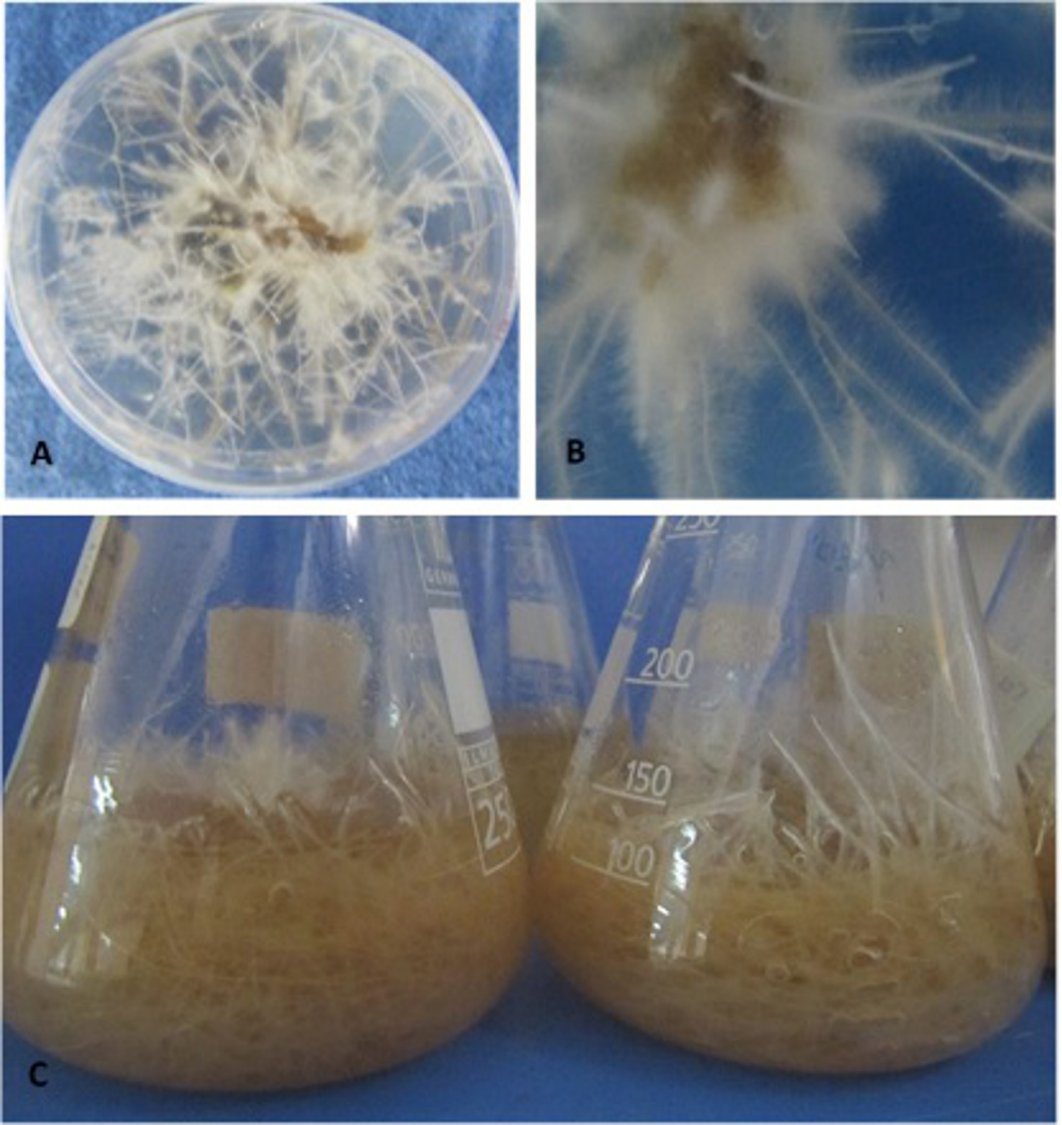
Formation and elongation of *N*. *tobacco* hairy roots at different periods after *A*. *rhizogenes* infection. Induction and propagation of hairy roots at the marginal edges of leaf explants grown on solid MS medium supplemented with 400 mg/L of cefotaxime and 150 mg/L of kanamycin at 25°C under a 16/8-h light/dark photoperiod at three weeks post-infection (A). High-magnification view of an explant showing the development of hairy roots (B). Growth of hairy roots cultivated in a 250-ml Erlenmeyer flask containing MS liquid medium without antibiotics for one or two months at 28°C in the dark with gentle shaking, with the medium being refreshed weekly (C).

Most of the *N*. *tobacco* hairy root clones were highly elongated and had a large number of branching lateral roots at 10 days after transformation (Fig 2A and 2B). The growth and branching of the hairy roots beginning at the site of *A*. *rhizogenes* inoculation was the first sign of successful transformation by this organism [48].

**Fig 3:**
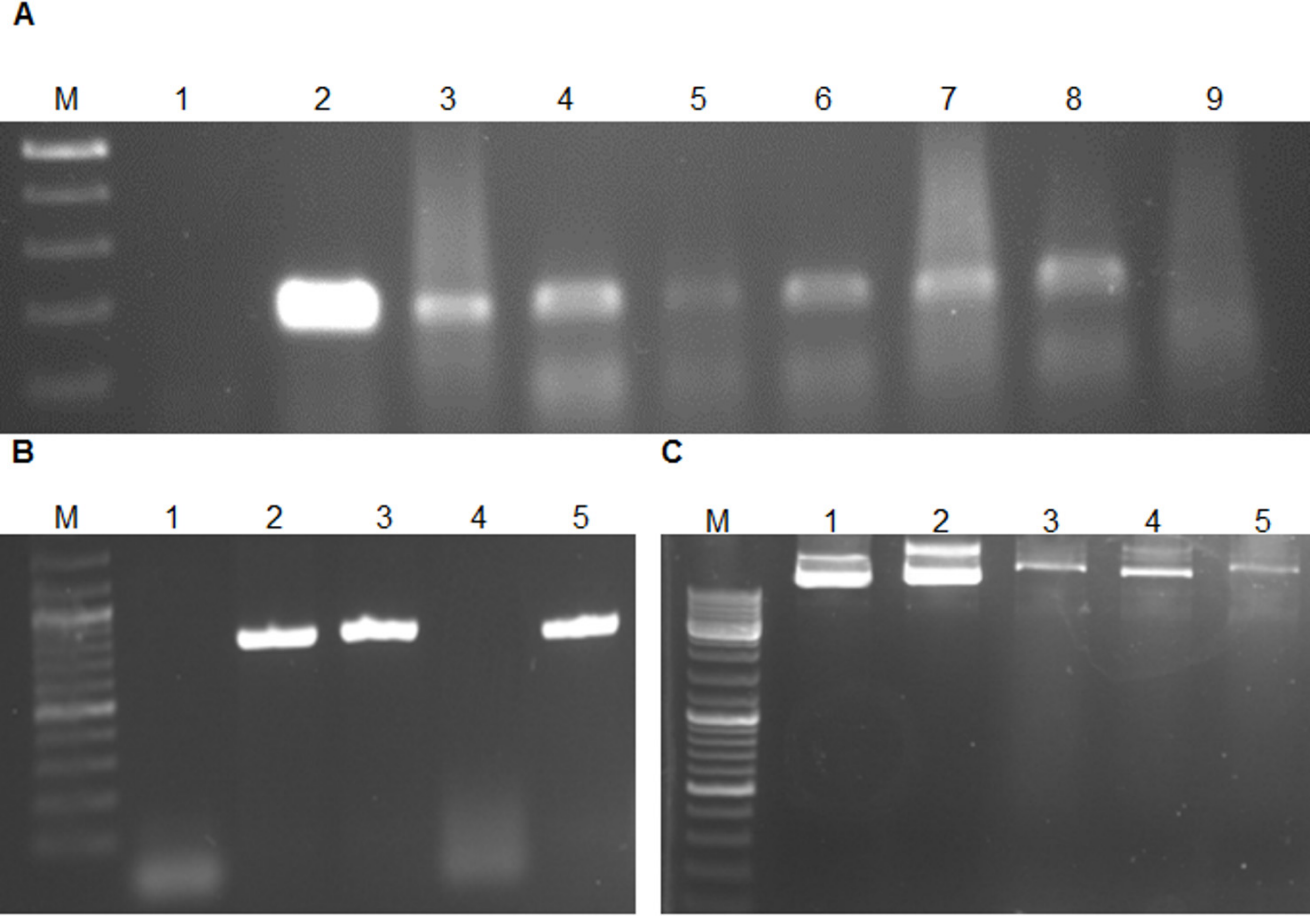
PCR amplification of a 194-bp fragment of *rolB* and *MAP30* using DNA and cDNA derived from *N*. *tobacco* hairy roots and the topological-activation activity of the KDEL-MAP30 fusion protein. Lane M, Ruler 1-kb DNA ladder Mix (Fermentas, Germany); Lane 2, PCR negative control (water); Lane 3, positive control (*A*. *rhizogenes* culture); Lanes 3–8, transgenic hairy root clones and Lane 9, nontransgenic hairy root clone (A). Lane 3, genomic DNA of transgenic hairy roots; Lane 4, genomic DNA of nontransgenic hairy roots; Lane 5, cDNA of transgenic hairy roots (B). Lane 1, plasmid DNA as a control; Lane 2, plasmid incubated with the total proteins extracted from nontransgenic hairy roots; Lanes 3–5, nicked and liner plasmid DNA after incubation with the total proteins extracted from transgenic hairy roots at 4, 25, and 37°C, respectively (C).

Some of the hairy roots might have escaped infection, remaining non-transformed [24]. Therefore, putative transformants that were grown on selection medium containing antibiotics were first examined for the absence of *A*. *rhizogenes* contamination using PCR with primers specific for *virG* [49], a bacterial gene that does not integrate into plant genomes. No specific fragment was amplified from these roots, whereas the expected *virG* fragment was detected in the positive control (data not shown). Hairy roots were rendered bacteria-free by transferring them weekly to fresh medium containing the antibiotics mentioned above. The presence of the *MAP30* CDS and the *rolB* in the genome of hairy roots clones and their expression of *MAP30* were confirmed by PCR and RT-PCR, respectively (Fig 3A and 3B). Our results confirmed the high transformation efficacy of *A*. *rhizogenes* (Fig 2A and 2B), allowing the rapid scaling-up of transgenic hairy root production (Fig 2C).

The hairy root clones exhibited insignificant phenotypically differences from those of non-transformed roots, which is an important parameter for scale-up (Fig 2C), according to the results of Alpizar et al. 2008 [49]. Fundamental to cultured hairy root systems is their ability to grow in the absence of plant-growth regulators [50,28], thus, the morphological and molecular characteristics of the hairy root clones were maintained over the long term (Fig 2C). The neoplastic (cancerous) roots produced by *A*. *rhizogenes* infection are characterized by a high growth rate, genetic stability and growth in hormone-free media [50,28]. In addition, because the hairy root culture medium was hormone-free, the possibility of hormone-induced chromosomal abnormalities causing genotypic instability was eliminated [25], which was reflected in the efficient production of functional rMAP30-KDEL by two month-old hairy root cultures (Fig 2C). Similar to suspended cells, hairy roots can be axenically cultured in a controlled environment that is suitable for the production of high-value pharmaceutical proteins under the requirements for good manufacturing practices [51,21].

### Protein extraction, purification and SDS-PAGE analysis

**Fig 4:**
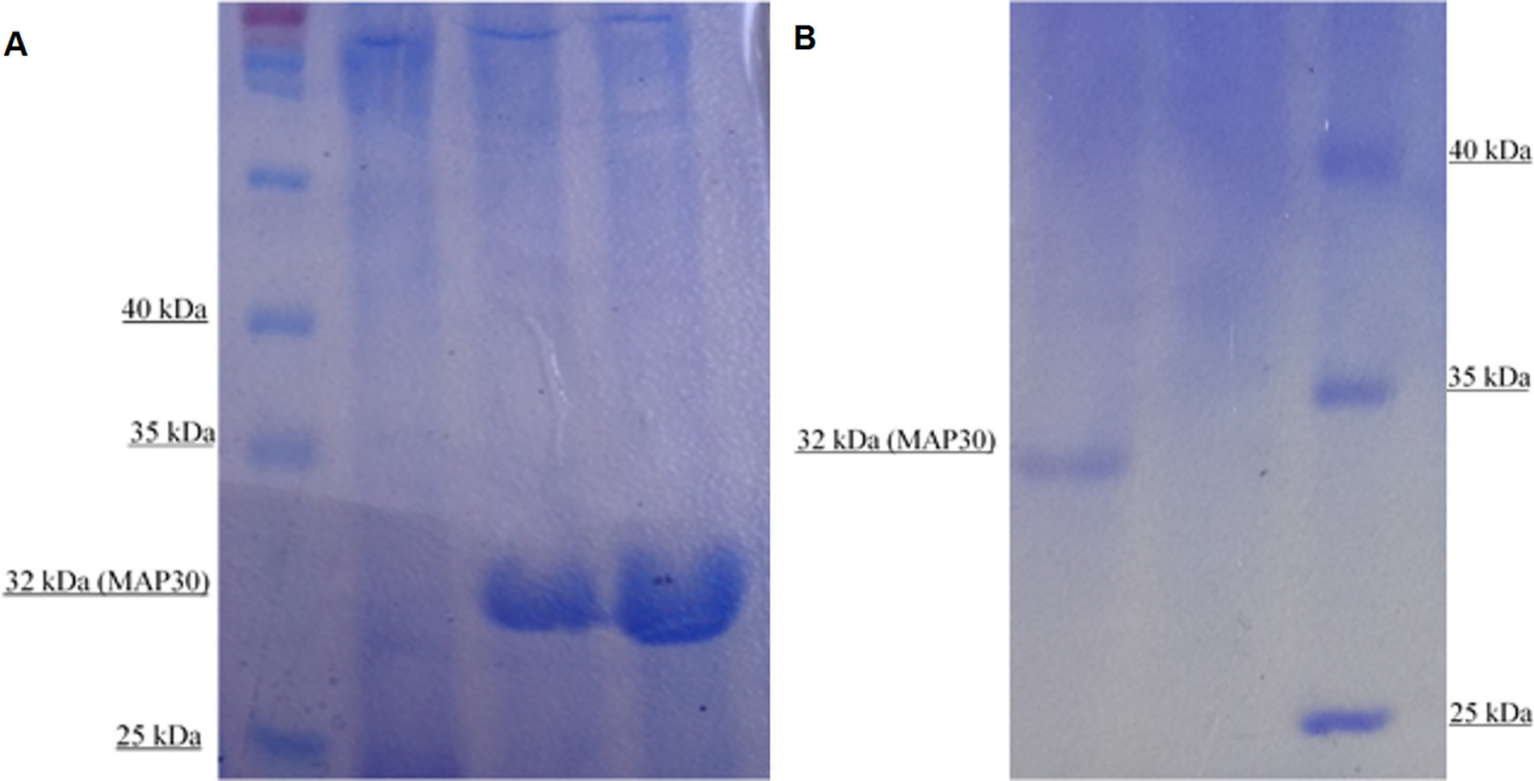
SDS-PAGE analysis of total extracted proteins that were boiled for 20 min and the protein purified under native conditions. Left to right: Lane 1, protein ladder (Thermo Scientific: #26616); Lane 2: total proteins extracted from nontransgenic hairy roots; Lanes 3–4, total proteins extracted from transgenic hairy root clones (A). Right to left: Lane 1, protein purified from a transgenic hairy root extract; Lane 2, protein purified from a nontransgenic hairy root extract; Lane 3: protein ladder (Thermo Scientific: #26616) (B). The presence of a band of approximately 32 kDa in the transgenic hairy root sample but not in the nontransgenic hairy root sample confirmed the expression of MAP30-KDEL by the transgenic hairy roots.

Because rMAP30-KDEL had previously showed thermal stability, the total proteins extracted from transgenic and non-transgenic hairy root clones were boiled for 20 min and then were analyzed by SDS-PAGE (Fig 4A). A major band of 32 kDa that correlated with rMAP30-KDEL was found in the transgenic hairy root clone samples but not in the non-transgenic hairy root samples (Fig 4A).

These results demonstrated that the rMAP30-KDEL expressed in transgenic hairy roots under the control of the strong CaMV 35S promoter comprised a significant fraction of the total proteins (Fig 4A). Many studies have shown that this system is extremely efficient in producing recombinant proteins. For example, GFP expressed under the control of the CaMV 35S promoter and enhancers represented 60% of the total soluble proteins secreted by *Brassica rapa* hairy roots [22].

To confirm the expression of rMAP30-KDEL in the transgenic hairy roots, the His-tagged protein was purified from the total extracted proteins using a Ni-NTA spin column under native conditions. A specific 32 kDa rMAP30-KDEL band was observed by SDS-PAGE, whereas this band was not purified from non-transgenic hairy root samples (Fig 4B). This result clearly demonstrated that rMAP30-KDEL had been expressed efficiently. Finally, rMAP30-KDEL was produced in hairy roots and was purified for use as an antimicrobial agent (data not shown).

### Comparison of the rMAP30-KDEL activities of the transgenic clones

**Fig 5:**
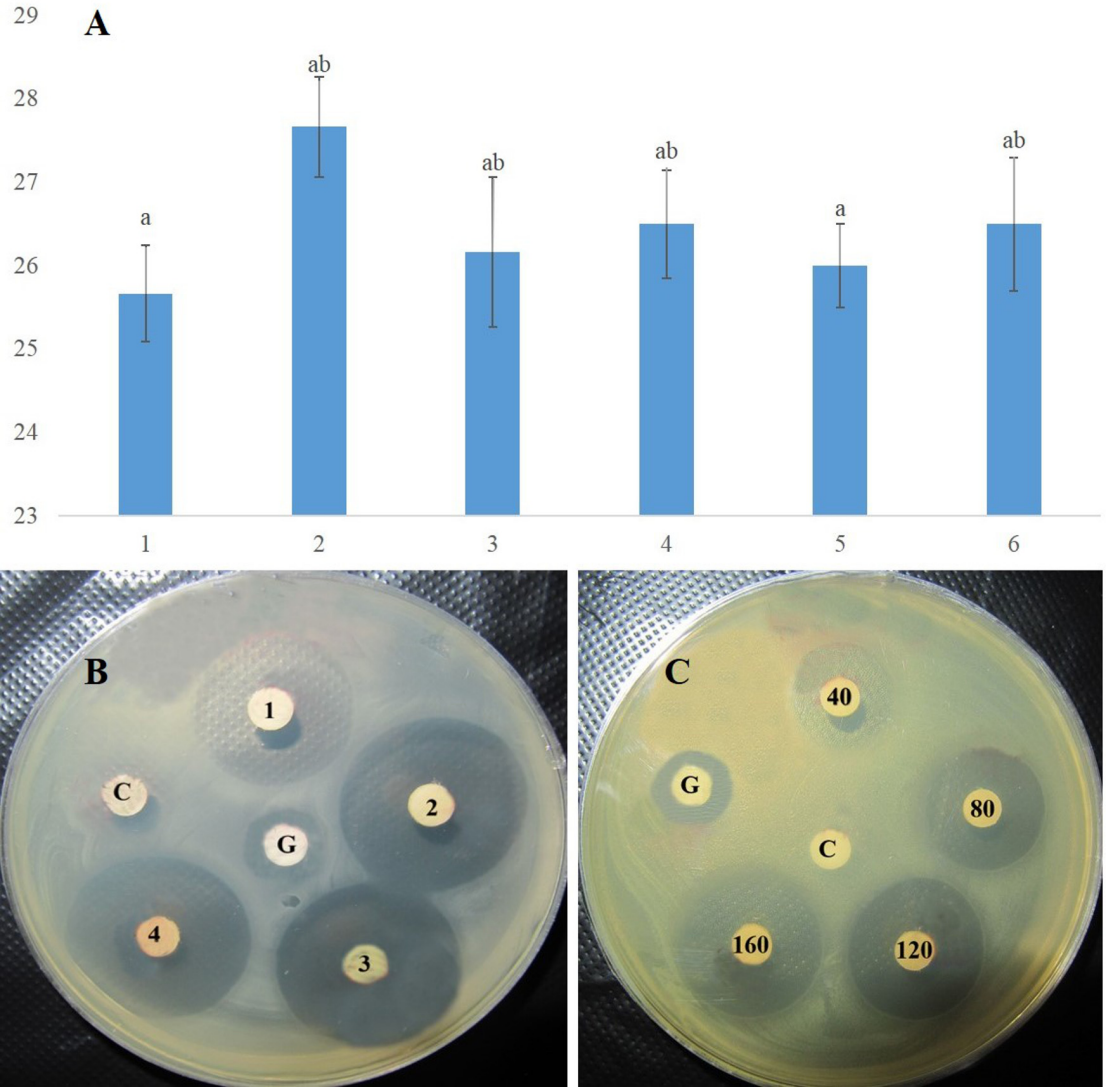
Antimicrobial activities of rMAP30-KDEL in samples of the hairy root clones. The antimicrobial activities of the total proteins (160 μg) derived from the transgenic hairy root clones were assayed using *E*. *coli* (A), which were compared using an inhibition-halo plate assay as well (B). Dose-dependence of the antimicrobial activity of rMAP30-KDEL was observed using 40, 80, 120, and 160 μg of total hairy root soluble proteins against *E*. *coli* for 16 h (C). G indicates gentamycin (10 μg/disc) and C indicates 160 μg of nontransgenic hairy root total proteins (control). The diameter of the inhibition zones in millimeters is shown on the x axis and the six transgenic hairy root clones are shown on the y axis. In all cases, a *P* value of ≤ 0.01 was considered significant.

The concentrations of the total proteins extracted from six transgenic hairy root clones were found to be 3.5, 3.7, 4.0, 4.0, 4.2, and 4.5 mg/mL in triplicate assays using the Bradford method. To compare the antimicrobial activities of the rMAP30-KDEL produced by the six clones, the effect of 160 μg of each total protein sample on *E*. *coli* growth was evaluated (Fig 5A and 5B).

Although the transgenic clones showed various high levels of antimicrobial activity, no significant differences were observed (Fig 5A and 5B). These results might be related to the high level of stability of the rMAP30-KDEL expression cassette and the efficient expression obtained by applying several strategies (Figs 4A and 1C), although the position and dosage of a transgene in a host genome can also affect the expression level and stability of the final product [28,32,52]. Finally, transgenic clone 2, which showed the highest level of antimicrobial activity (Fig 5A and 5B), was chosen for scaling-up under controlled conditions for further studies (Fig 2C).

### DNase activity of rMAP30-KDEL

Previous studies showed that MAP30 possesses topological-inactivation activity toward viral DNAs and plasmids [53–54]. Total protein samples (40 μg) containing the recombinant fusion protein 6-His-MAP30-KDEL exhibited topological-inactivation ability, nicking a supercoiled double-stranded plasmid and converting it to the linear form in a 2-h incubation, whereas the non-transgenic hairy root protein extracts did not have either of these effects (Fig 3C). In addition, the effect of the DNase-like activity of this protein on genomic DNA was the production of approximately 700-bp fragments (data not shown). These results were obtained at temperatures of 4, 25, and 37°C (Fig 3C). Therefore, the DNase activity of MAP30-KDEL was independent of temperature (Fig 3C). Topological inactivation is the key biological activity of MAP30 [37], and other RIPs show partial nuclease activity that results in the cleavage of supercoiled DNA [37], single-stranded DNA [55], and both double-stranded and supercoiled DNA [53,56].

Although it has been reported that a minor conformational modification of the 3-D structure of RIPs might result in a major change in their biological activities [57], the results obtained in this study demonstrated that rMAP30 fused with a His-tag and a KDEL peptide at the N- and C-terminus, respectively, retained its native biological properties, which were topological-inactivation and antimicrobial activities (Figs 3C and 5). Other researchers previously found that fused tags such as the His-tag had no effect on rMAP30 activity [35,41]. Recently, functional recombinant MAP30 protein fused with the antiviral cationic peptides protegrin-1 and plectasin that protect against dengue virus infection [7], the anticancer peptides tachiplicin I and latarcin 1 [41], and a cell-penetrating peptide that acts against cancer cells [8] have been produced. The activity of MAP30 appears not to be much affected by the fusions because proteolytic fragments of MAP30 exhibit anti-HIV and anti-tumor activity [35,58].

### Antimicrobial activity of rMAP30-KDEL

The antimicrobial activity of the total proteins extracted from hairy root clone 2 was assayed using various microorganisms (Table 2). The results of the disc-diffusion assay demonstrated that rMAP30-KDEL had strong antimicrobial activity against all of the evaluated animal- and plant-infecting bacteria (including important plant pathogens that cause severe diseases, such as wilt, crown gall, leaf spot, and soft rot) and against fungi (Table 2). Other researchers have demonstrated the antibacterial, antiviral and antifungal activities of type I RIPs [59].

**Table 2:** Duncan’s multiple range test of the differences in the antimicrobial activities against some bacteria and fungi of MAP30-KDEL applied at different concentrations (*P* ≤ 0.01).

Microorganism	Average of inhibition zone (mm)					
	N	40 μg	80 μg	120 μg	160 μg	Antibiotic
*E*. *coli*	-	22±0.41^mnops^	24.32±0.42^hijklm^	26.59±0.42^efghw^	28±0.38^cdefg^	11±0.42^uvw^
*S*. *aureus*	-	24.64±0.40^hijkl^	27.83±0.40^defg^	30.62±0.41^ab^	32±0.32^a^	12±0.31^uvw^
*P*. *aeruginosa*	-	22.33±0.34^lmnops^	25±0.38^hijk^	28±0.37^cdefg^	29±0.42^bcde^	15.33±0.35^t^
*B*. *subtilis*	-	19.35±0.34^qr^	22.31±0.39^lmnop^	24.68±0.51^hijkl^	24.65±0.41^hijkl^	12±0.46^uv^
*C*. *albicans*	-	26±0.32^fghi^	28.33±0.31^bcdef^	29.33±0.52^bcd^	30.68±0.51^ab^	12±0.31^uvw^
*L*. *mesenteroides*	-	25±0.43^hijk^	28.5±0.36^bcde^	29.66±0.46^bcd^	30.33±0.34^abc^	11±0.42^uvw^
*A*. *niger*	-	20.61±0.42^opqr^	24±0.35^ijklm^	25.66±0.43^ghij^	27.7±0.45^defg^	11±0.3^uvw^
*S*. *epidermidis *	-	13±0.39^u^	16.36±0.38^t^	18.33±0.47^r^	18.67±0.32^r^	8±0.31^x^
*S*. *typhi *	-	20±0.35^pqr^	22.83±0.46^klmno^	23.32±0.43^klmn^	22.83±0.51^klmno^	12±0.31^uvw^
*X*. *citri*	-	18.63±0.45^r^	20±0.36^pqr^	22±0.29^mnops^	25±0.36^hijk^	15.33±0.44^t^
*R*. *solanacearum*	-	9±0.3^x^	10.33±0.47^vw^	10.33±0.42^vw^	12.61±0.36^uv^	11±0.44^uvw^
*P*. *syringae*	-	18.31±0.36^r^	21.34±0.34^nopq^	23.67±0.52^ijklmn^	23.64±0.46^ijklmn^	11±0.31^uvw^

The control antibiotics used (10 μg/disc) that are specific for Gram-negative, Gram-positive bacteria and fungi were gentamycin, ampicillin, and ketoconazole, respectively.—No growth inhibition; *N*, nontransgenic hairy roots.

The Gram-negative bacteria most susceptible to rMAP30-KDEL were *P*. *aeruginosa* and *E*. *coli*, whereas the most susceptible Gram-positive bacterium and fungus were *S*. *aureus* and *C*. *albicans*, respectively (Table 2). The results showed that rMAP30-KDEL could control the growth of different pathogenic microorganisms at very low concentrations (Table 2). One study has shown that as little as 0.5 μg of two types of purified I RIPs, ME1 and ME2 derived from *Mirabilis expansa* roots, had antimicrobial activity after 24 h of incubation [59]. Vivanco et al. 1999 have demonstrated the growth-inhibitory activity of total storage-root proteins applied at different concentrations (in micrograms) against various bacteria and fungi using an inhibition-halo plate assay [59].

Arazi et al. 2002 have also demonstrated that rMAP30 expressed in cucurbit plants, including squash, cucumber, melon, watermelon, and pumpkin, showed antiviral and antimicrobial activities at very low concentrations (0.2 nM for HIV-1 and 0.79 μg/mL for some bacteria) [35]. They demonstrated that rMAP30 is an effective agent for defense against various pathogens, including *S*. *aureus*, *E*. *coli*, *C*. *albicans*, and *A*. *fumigatus* [35]. The dose-dependency of the antimicrobial activity of rMAP30-KDEL was observed when 40, 80, 120, and 160 μg of total hairy root soluble proteins were applied for 16 h (Fig 5C), and the zones of inhibition persisted at 6 weeks after inoculation. Vivanco et al. 1999 established that inhibition zones were produced when 6.5 μg to 50 μg of the total root soluble proteins of *M*. *expansa* were applied to various microorganisms, that the size of the zones of inhibition were dose-dependent and that the antifungal activity persisted at 5 weeks after treatment [59]. MAP30 not only controlled the de novo infection of cells but also inhibited viral replication in previously infected cells [35,42]. MAP30 is an efficient anti-cancer and antiviral protein that specifically affects tumor cells and viral-infected cells and to date has not been found to be toxic to uninfected normal cells [36–37,41].

Recently, MAP30 was shown to have antimicrobial activity against *S*. *aureus*, *B*. *subtilis*, and *E*. *coli*, as well as *C*. *albicans* [42]. It is notable that *C*. *albicans* is regarded as the leading cause of invasive microbial disease in patients with several syndromes [35]. The effects of nanoencapsulated MAP30 in treating a *C*. *albicans* infection confirmed the existence of a multisystemic disease and showed that its strong detoxification and antimicrobial activities were due to its biocompatibility; therefore, drug delivery systems for MAP30 are promising candidates for therapeutic applications [42]. Because PBRPEs provide target proteins with suitable PTMs that are free of microbial toxins or animal pathogens [12], the use of plants for large-scale protein synthesis is gaining wider acceptance [17,21–22,25]. The HR model of recombinant-protein production has also been demonstrated to be safer and to be a valuable alternative to whole-plant molecular farming systems, traditional production systems based on microbial fermentation, insect and mammalian cell cultures, and transgenic animals in terms of cost, scalability, product safety and the authenticity of the products [3,19].

### Strategies applied for the efficient expression of rMAP30

The results obtained demonstrated for the first time that the multifunctional plant protein rMAP30-KDEL could be produced in *N*. *tobacco* hairy roots (Figs 3C, 4A, 4B, 5A and 5B). Even proteins that are very toxic to animals [3,12,22] and several other antibacterial proteins have been successfully produced using this system [21]. Studies have shown that the high-level production of a biologically active recombinant protein using this system first depends on the elements of the gene cassette, such as a strong promoter and proper polyadenylation site, which are often derived from the 35S gene of cauliflower mosaic virus (CaMV) (Fig 1C). Widely used polyadenylation sites include those of the CaMV 35S transcript and the Agrobacterium nos transcript [3,60]. The second important factor for this property is codon-usage compatibility between the target-gene sequence and the genome of the expression host [61]. Recently, MAP30 was expressed from the genomic DNA of *M*. *charantia* in an *E*. *coli* prokaryotic system [40–41]. The efficacy of this strategy of expressing a eukaryotic gene in a prokaryotic expression host without codon optimization was limited, which is generally caused by transgenes with various codon biases including disfavored codons and resulting in frame shifting [3]. Thus, eukaryotic expression hosts have been established for the production of complex proteins that do not properly fold in bacterial hosts [61–62]. In this study, the *rMAP30-KDEL* CDS was codon-optimized for expression in *N*. *tobacum* hairy roots, and the results demonstrated that the resultant protein was efficiently expressed in all of the transgenic hairy root clones and was an effective antimicrobial and topological-inactivation agent at low concentrations (Figs 3C and 5B; Table 2). It is accepted that codon bias plays a fundamental role in heterologous gene expression and that the lack of codon optimization can limit the level of gene expression due to the deficiency of accessible tRNAs in the host, hampering the elongation of the target peptide or resulting in incomplete translation [63,64]. Therefore, another explanation for the strong antimicrobial activity of rMAP30-KDEL might the codon optimization of its CDS based on the host expression system used (Figs 1C and 5; Table 2).

The third and the most important factor affecting the yield of recombinant proteins is subcellular targeting, which affects the folding, assembly, and PTM processes [3]. A protein with a functional KDEL tetrapeptide would be retrieved from the Golgi apparatus via retrograde transport to the ER lumen [31]. Therefore, the KDEL tetrapeptide ER-retention signal was fused to the C-terminus of MAP30 (Fig 1C) to target the recombinant protein to the ER lumen, which is a subcellular location that is safe from host proteinases and includes chaperone and glycosylation systems for proper folding and stability and the addition of suitable glycan groups, respectively [3]. The yield of proteins expressed via the ER-retention technique is greater than that of proteins secreted into the culture medium [3]. For example, the yield of an antibody was increased when it was retained in the ER lumen via its fusion with a C-terminal KDEL tetrapeptide [31]. In addition, protein glycosylation occurs only in the endomembrane system, and this modification is required for the proper functioning of many proteins of human origin [3].

In nature, the ER-targeting of an endogenously synthesized inactive precursor toxin appears to be the mechanism by which *R*. *communis* L. cells avoid its toxicity [65,66]. Although Wang et al. 2014 expressed MAP30 in *Pichia pastoris* using the genomic DNA of *M*. *charantia* without codon optimization, they could not achieve an effective yield, which might be related to not using a subcellular targeting strategy and the degradation of the non-optimized gene [66]. In contrast, hairy roots showed more efficient production of MAP30 (Fig 4) with strong activity (Table 2; Fig 5). These results might be related to the use of the strategies of codon optimization based on the host genome and the fusion of KDEL ER-retention signal at the C-terminus of MAP30.

### Thermal and pH stability and their interactions

**Fig 6:**
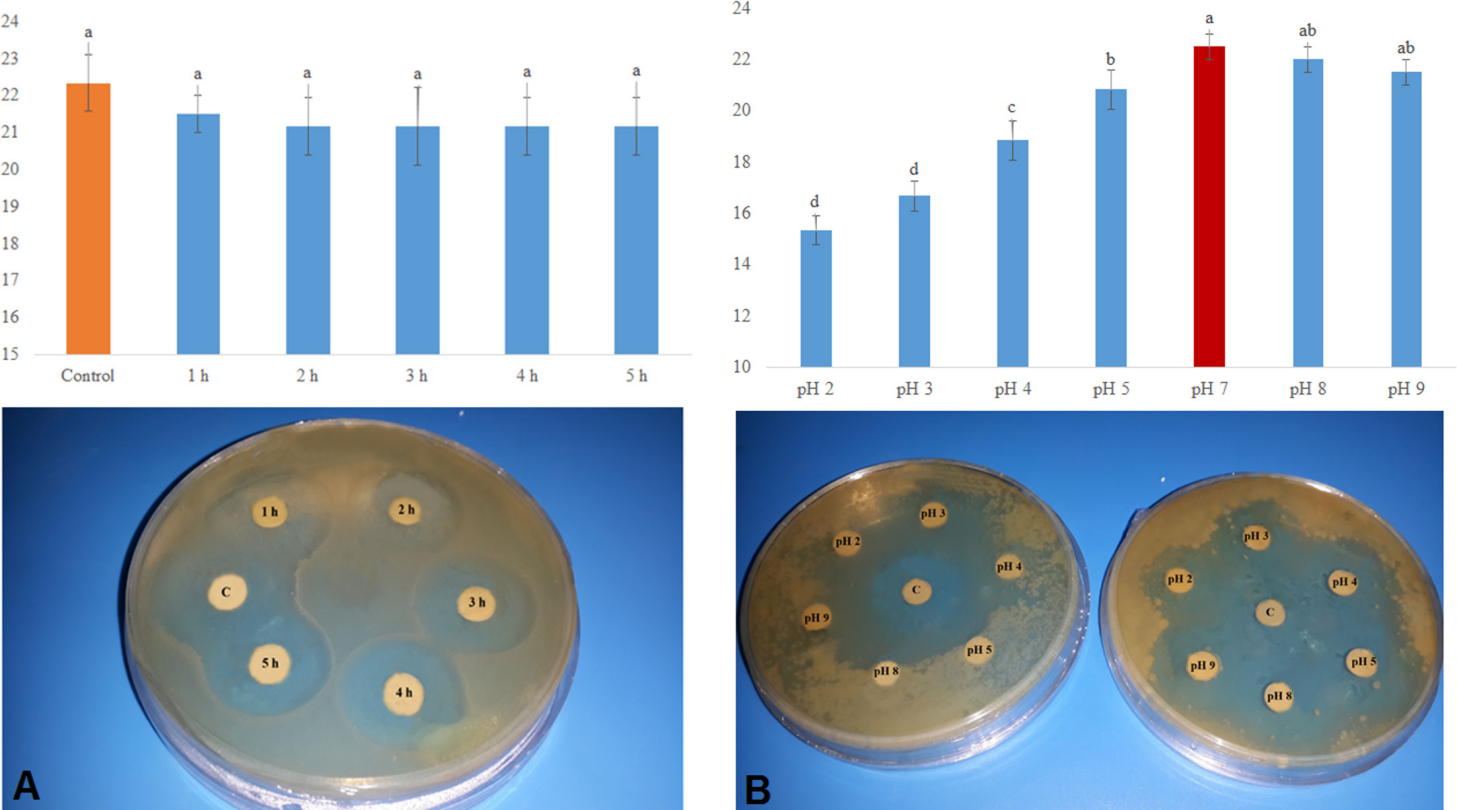
Thermal and pH stability of rMAP30-KDEL. The high level of thermal stability of rMAP30-KDEL (160 μg) after 1, 2, 3, 4, and 5 hours of boiling was shown by its antibacterial activity against *E*. *coli* not being significantly affected by the length of the heating period (A). Acidic pH values inhibited the activity of rMAP30-KDEL, whereas it had a similar level of activity at basic pH values as at physiological pH. The left panel shows a control plate containing solutions (without protein) with the tested acidic and basic pH values, and the right panel shows the total proteins adjusted to the tested pH values (B). C shows untreated transgenic root total proteins. In all cases, a *P* value of ≤ 0.01 was considered significant.

rMAP30-KDEL showed a high level of thermal stability, with its antibacterial activity against *E*. *coli* not being significantly affected by the length of the heating period (Fig 6A); the observed activity level was stable for up to 6 weeks post-treatment. Bitter melon, a source of MAP30, has been used in various Asian and African herbal medicine systems for many years as an edible remedy for a variety of ailments, particularly stomach complaints and diabetes [66], and *M*. *expansa* storage roots have been used as a food source of RIPs [59]. Presumably, the activity of MAP30 and other RIPs might resist cooking and roasting processes. Some types of RIPs, such as PAP [67], ME1 and ME2 [59], gelonin [68], and ricin [56], resist denaturation due to boiling, maintaining their activities.

rMAP30-KDEL exhibited a range of activities at different pH values (Fig 6B). Though this protein had the highest level of activity at pH 7 and the lowest level of activity at pH 4, its activity level at the basic pH values of 8 and 9 were not significantly different from that at pH 7. The basic pH values had a less disruptive effect on rMAP30-KDEL activity compared with that of the acidic pH values, such as 2, 3, 4, and 5, which had a significantly disruptive effect (Fig 6B). Changing the pH value affects a protein by altering the electrostatic properties of the amino-acid side chains and the protein surfaces [69], which irreversibly alters the salt bridges or ionic bonds between positively and negatively charged side chains, eliminating their ionic attractions and resulting in protein unfolding [69].

**Fig 7:**
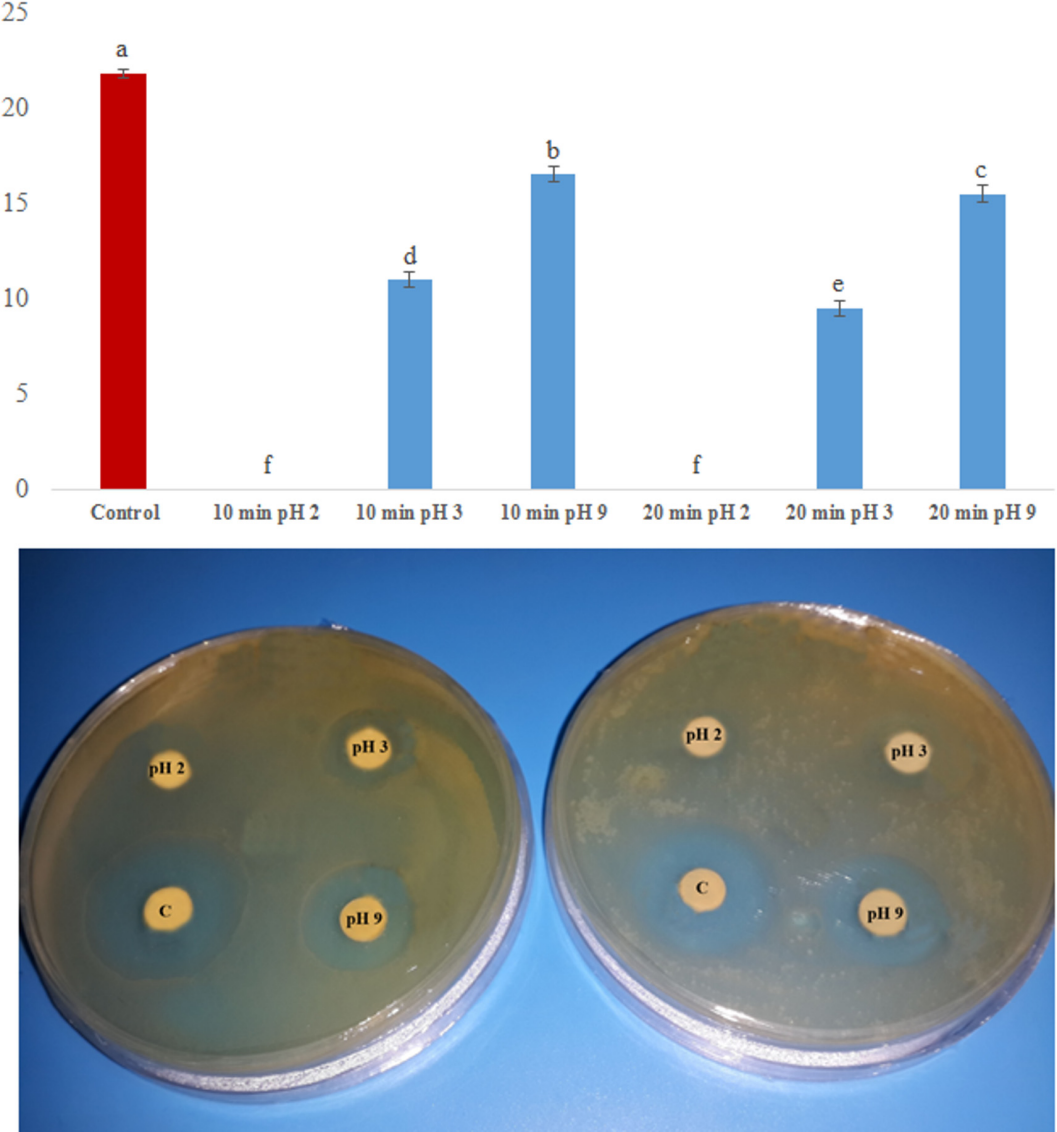
Synergic effect of pH and temperature on the activity of rMAP30-KDEL. Solutions of the total proteins of transgenic hairy roots (160 μg) were adjusted to the acidic pH values of 2 or 3 or the basic pH value of 9 and were incubated at 80°C for 10 (left plate) or 20 min (right plate). The level of antimicrobial activity against *E*. *coli* was determined. C shows the effect of untreated transgenic root total proteins. In all cases, a *P* value of ≤ 0.01 was considered significant.

In addition to causing such changes, changing the pH value disrupts the hydrogen bonds between the side chains of amino acids, which changes the shape of a protein [70]. These changes will affect and might disrupt the secondary and tertiary structures of a protein, changing its shape [69] and causing a decrease or loss of its functionality [Fig 6B]. The results of this study showed that changes in the pH value were more disruptive to the activity of MAP30-KDEL than were changes in temperature [Fig 6A and 6B]. High temperature and a low pH value had a negatively synergic effect on MAP30-KDEL activity because MAP30-KDEL had no activity after being heated to 80°C for 20 min at pH 2, and its activity was reduced to half that observed after heating at pH 3 for the same period (Fig 7). However, heating at pH 9 had a less disruptive effect on its activity (Fig 7).

Similar to the case for MAP30, when the A chain of ricin was denatured by boiling it for 10 min, its RNA N-glycosidase activity was entirely eliminated, although its capacity to cleave supercoiled DNA persisted, albeit it at a decreased level compared with that of the nondenatured A chain of ricin [56]. These results provide further support for the hypothesis that these two types of enzymatic properties of one RIP molecule might not be closely related [56]. For example, the DNA-damaging activity of gelonin was not eliminated by boiling and this activity is thought to be independent of its ribosome-inactivating activity [68].

### Level of identity of RIPs and identification of their conserved motifs and domains

Limited proteolysis yielded fragments of the RIPs MAP30 and GAP31 that retained full HIV-integrase inhibition and HIV-LTR topological-inactivation activities, as well as tumor cell-growth inhibitory activity at very low concentrations ranging from 0.2 to 0.4 nM but did not retain their ribosome-inactivation activity [53]. These RIPs display a variety of antimicrobial activities and broad-spectrum antiviral properties against both human and animal pathogens [71] and understanding their exact mechanisms of action in depth would be beneficial [72]. It has been stated that as few as one RIP molecule per cell could entirely inhibit protein synthesis [33].

**Fig 8:**
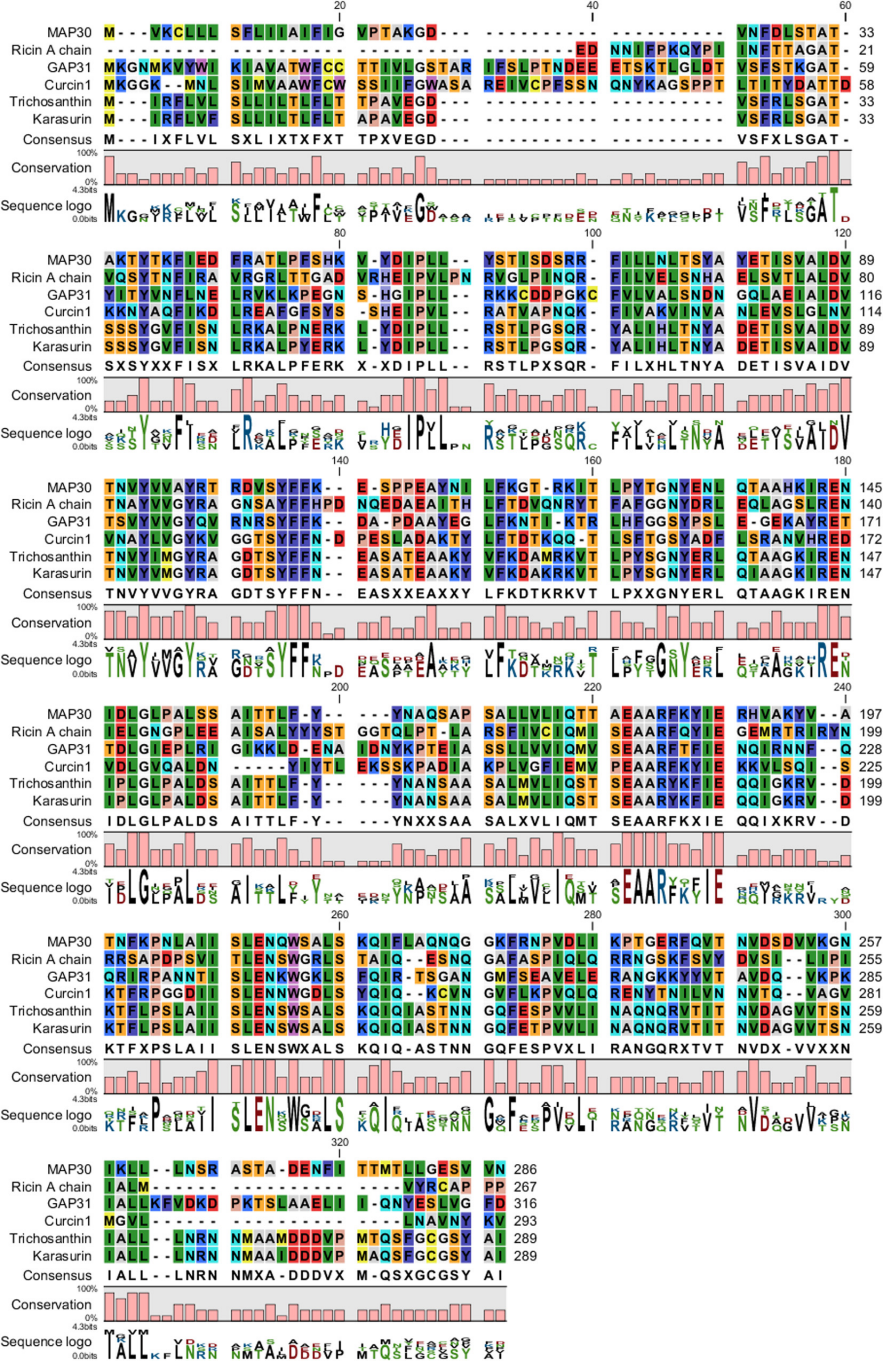
Multiple alignment of the amino-acid sequences of RIPs from the following plant species: the MAP30 from *M*. *charantia*, A chain of ricin from *R*. *communis*, trichosanthin and karasurin from *T*. *kirilowii*, GAP31 from *S*. *multiflora*, and curcin 1 from *J*. *curcas* performed using CLC Main Workbench 5 software (Qiagen). Conserved amino acids were found in regions with a high level of identity, particularly in the central region.

**Fig 9:**
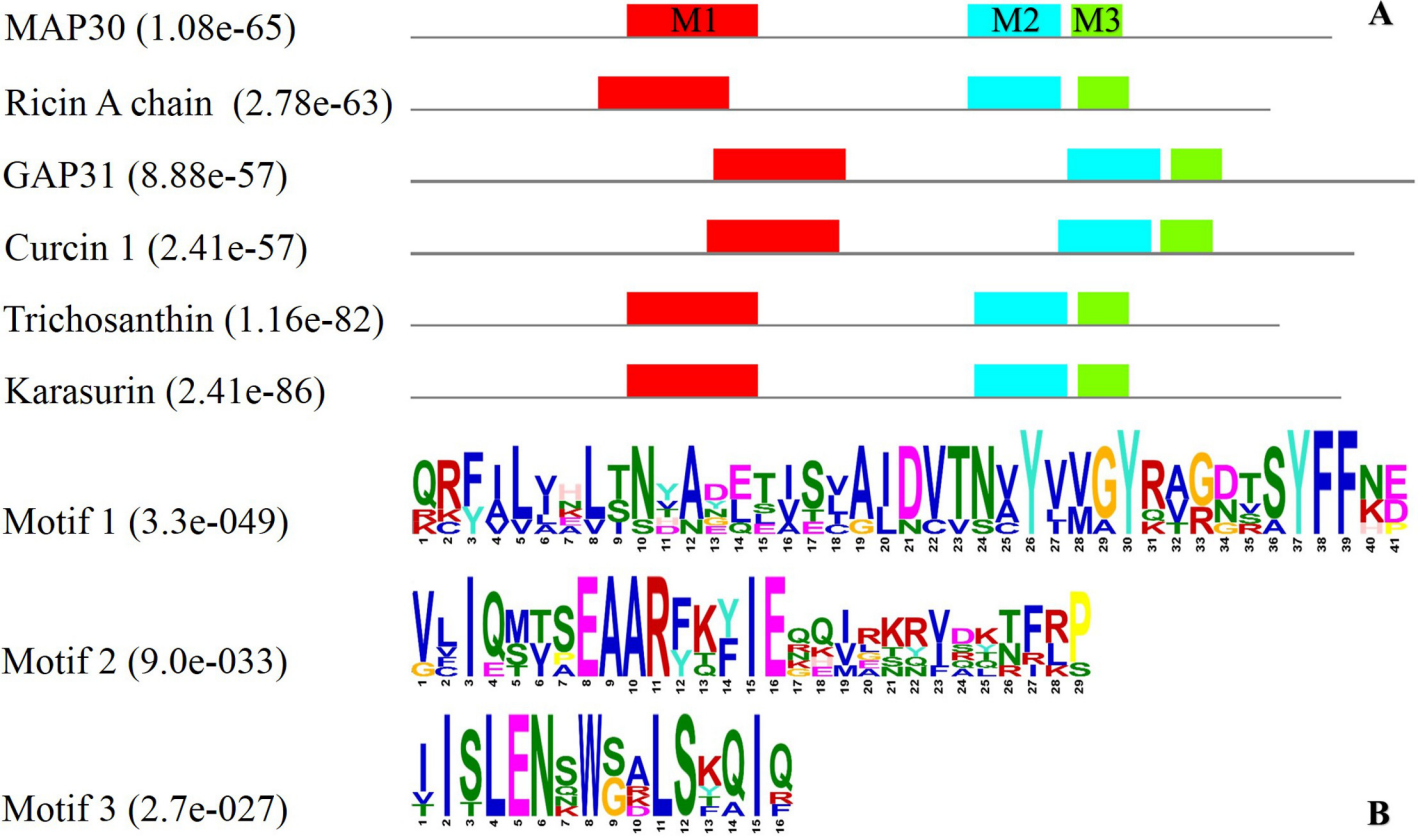
Identification of the conserved motifs in RIPs from the following plant species: the MAP30 from *M*. *charantia*, A chain of ricin from *R*. *communis*, trichosanthin and karasurin from *T*. *kirilowii*, GAP31 from *S*. *multiflora*, and curcin 1 from *J*. *curcas* conducted using MEME. Motifs 1, 2, and 3 are shown in red, blue, and green, respectively, and the *P* values are reported in parentheses (A). The consensus sequences of these three RIP motifs were determined, and the *E* values are reported in parentheses (B).

Conserved residues that are potentially associated with their functions were found in the RIPs from different plant species (Figs 8 and 9). BLAST-P analysis and alignment of the RIP amino-acid sequences were performed, which revealed conserved amino acids in regions with a high level of identity (of approximately 30 to 90 percent), particularly in the central region (Fig 8).

Comparison of the sequences of trichosanthin and karasurin from *T*. *kirilowii* and that of MAP30 showed that the level of intraspecies identity of these RIPs was 93 percent and that their level of interspecies identity was greater than 50 percent (Table 3). Analysis of the structural and functional organization of the regions of the anti-HIV and anti-tumor proteins MAP30 and GAP31 by limited proteolysis with endopeptidases showed that the central regions of these proteins were resistant to proteolysis, whereas the N- and C-termini were susceptible to proteolysis [53]. The results also indicated that the thermal and pH stability of MAP30-KDEL might be related to the resistance of its central region (Figs 6 and 7).

**Table 3:** Percentage of identity among the best known RIPs.

RIPs	Ricin A chain	GAP31	Curcin 1	Trichosanthin	Karasurin
MAP30	31	36	30	56	54
Ricin A chain		34	29	36	36
GAP31			32	34	36
Curcin 1				30	31
Trichosanthin					93

The motifs and domains of the best-known RIPs were analyzed to find the conserved regions (Figs 1A, 1B, 9A and 9B; Table 4]. The three highly conserved motifs found in the best-known type I RIPs were compared with those of a type II, the RIP A chain of ricin (Fig 9A and 9B; Table 4). Studies have demonstrated that the antiviral and anti-tumor activities of MAP30 and GAP31 are independent of their ribosome-inactivation activity and that the N-terminal 10 amino acids of both MAP30 and GAP31 as well as the C-terminal 76 amino acids of MAP30 and the C-terminal 56 amino acids of GAP31 are not required for their antiviral and anti-tumor activities but appeared to be required for their ribosome-inactivation activity [37,68,35].

**Table 4:** Identification of the conserved motifs in RIPs using MEME. The amino acids comprising each motif were determined.

RIPs	Motif 1	Motif 2	Motif 3
MAP30	68–108 (1.05e-35)	174–202 (1.26e-25)	206–221 (3.67e-17)
Ricin A chain	59–99 (6.08e-37)	174–202 (9.26e-24)	208–223 (3.03e-15)
GAP31	95–135 (3.59e-29)	205–233 (1.19e-24)	237–252 (8.96e-16)
Curcin 1	93–133 (7.80e-23)	202–230 (1.85e-23)	234–249 (9.07e-19)
Trichosanthin	68–108 (1.55e-45)	176–204 (3.19e-30)	208–223 (8.34e-20)
Karasurin	68–108 (1.56e-48)	176–204 (4.80–31)	208–223 (8.34e-20)

The *P* values are presented in parentheses.

The N- and C-termini of the RIPs did not contain conserved motifs (Fig 9A), which is consistent with these regions being unrelated to the ribosome-inactivation activity, a property that might not be exhibited by all RIPs. The three conserved motifs in the central region of the RIPs (Fig 9A and 9B) were extended to a create a critical domain [Fig 1A and 1B] for the antiviral, antimicrobial and anti-tumor activities of most type I and type II RIPs (Fig 9A and 9B).

These results also demonstrated that the topological-inactivation, antimicrobial and antiviral activities of the evaluated RIPs are independent of their ribosome-inactivation activity. Type I RIPs, such as trichosanthin, karasurin, and MAP30, had highly similar motif structures compared with those of the type II RIP analyzed, the ricin chain A, indicating that they might have the same mechanism of action (Fig 9A and 9B). Wang et al. 1999a demonstrated that the overall folding of MAP30 and the A chain of ricin are essentially the same and that the secondary structure and β-sheet topology of MAP30 are very similar to those of the A chain of ricin [73].

Interestingly, GAP31 and curcin 1 differed from the other RIPs by having their motifs in slightly shifted positions, which could affect their structures and functions (Fig 9A). These results indicated that GAP31 might act through mechanisms more similar to those of ricin than to those of MAP30. In addition, Arazi et al. 2002 showed that MAP30 and GAP31 exhibited different patterns of dose-dependent inhibition of HSV (herpes simplex virus), with MAP30 being more effective than GAP31 [35]. Whereas many studies have focused on anti-HIV peptides and lectins and their ability to inhibit the growth of bacteria and fungi has long been known, MAP30 is not yet a perfect clinical drug due to various unsolved issues [74,42].

The mechanisms of action of these macromolecules might be the formation of ion channels in the microbial membrane [75] or the competitive inhibition of the adhesion of microbial proteins to the polysaccharide receptors of the host [76]. Lectins such as MAP30, GAP31, and jacalin most likely inhibit viral proliferation by inhibiting the interaction of viruses with critical host-cell components [37]. However, to reach the ribosomes, RIPs must penetrate the target cell, which is difficult for type I RIPs due to their deficiency in sugar-binding activity [71]. These molecules can enter cells, most likely due to their interaction with the phospholipids in the cell membrane; however, their exact mechanism of entry remains unclear [71]. Certain anatomical features such as gaps, natural openings, and damaged tissue may facilitate the penetration of these RIPs; for example, barley RIP can penetrate cells through gaps in the plasma membrane [77]. In vivo, RIPs may act synergistically with other defense-related proteins, such as chitinases [78] and β-1,3 glucanases [79]; the latter proteins may degrade fungal-cell walls, thus facilitating the entrance of RIPs into these cells.

### Effect of the amino-acid composition of RIPs on their thermal stability

Bitter melon is a thermophilic plant that is compatible with tropical, hot, and humid weather conditions [35,53] that might prove to be a suitable source of various thermophilic proteins. The initial scans of the amino-acid compositions of several RIPs invariably showed a high content of several of Ala, Val, Ile, and Leu, whereas their contents of aromatic amino acids, including Phe, Trp, His, and Tyr, were more varied (Table 5). In addition, the potentially attractive features of the stability of MAP30-KDEL at high temperatures (Fig 6A) and its resistance to denaturants such as acids and (Figs 6B and 7] are notable, which is in agreement with the results of Ikai 1980 [80]. Of the 20 amino acids, Asn, Gln, Met and Cys are classified as thermolabile due to their tendency to undergo deamidation or oxidation at high temperatures [81].

**Table 5:** Distribution of the amino acids of RIPs determined using ProtParam.

Amino acid	MAP30	Ricin A chain	GAP31	Curcin 1	Trichosanthin	Karasurin
Ala	9.10	9.00	7.30	8.20	10.70	11.40
Arg	4.20	7.90	4.40	2.70	5.60	4.80
Asn	6.30	6.70	6.30	7.20	7.00	6.20
Asp	4.20	3.40	4.70	4.40	3.30	4.20
Cys	0.30	0.70	1.30	1.00	1.50	0.30
Gln	2.80	4.90	2.80	4.10	3.70	3.80
Glu	4.20	6.00	6.60	4.10	5.20	3.80
Gly	3.50	6.40	6.30	6.10	3.30	4.80
His	1.00	1.50	0.60	0.70	0.40	0.30
Ile	8.00	8.20	7.30	6.10	8.10	8.00
Leu	10.80	8.20	8.90	8.20	12.20	10.70
Lys	5.90	0.70	8.20	7.20	3.00	3.80
Met	0.70	1.10	1.30	1.70	1.90	1.70
Phe	5.90	4.90	5.10	5.50	4.10	4.50
Pro	3.80	5.60	3.50	3.80	3.30	3.50
Ser	7.30	6.70	6.30	7.50	9.60	9.30
Thr	9.10	6.40	7.00	5.50	7.80	6.60
Trp	0.30	0.40	0.90	1.40	0.40	0.30
Tyr	5.20	5.20	4.10	5.10	4.40	5.20
Val	7.00	6.00	7.00	9.60	4.40	6.60

Table 5 shows that the frequencies of Met, Cys, and Gln in the analyzed RIPs differed considerably. Tyr and Arg occurred more frequently in the thermophilic proteins, whereas Cys occurred less frequently (Table 5). The contents of the thermolabile residue Cys were substantially decreased in the thermophilic proteins compared with those of their mesophilic homologs, whereas those of Tyr and Arg were dramatically increased [82]. Due to their large side chains, Tyr and Arg may participate in both long-range and short-range interactions [81]. The guanidinium group of Arg can form salt bridges due to its short side chain, whereas Ser interacts mostly at a short range [83]. The key spots for binding at protein interfaces have been shown to be rich in Tyr, Trp, and Arg [84]. Therefore, it appears that Tyr and Arg play a similar role in protein binding and in the conformational maintenance of proteins at high temperatures, which affect their stability [81].

### Effect of hydrophobicity on the stability of RIPs

A low grand average of hydropathicity (GRAVY) value and a high aliphatic index are two major properties of thermophilic proteins [85]. The RIPs had very high aliphatic indices and very low GRAVY values, comparable to those of other thermophilic proteins (Table 6). With the rapid increase in the structural information available for proteins, it is becoming clear that hydrophobicity is the main driving force for protein folding [85].

**Table 6:** Biophysical properties of RIPs determined using ProtParam.

Biophysical properties	MAP30	Ricin A chain	GAP31	Curcin 1	Trichosanthin	Karasurin
Negatively charge	24	25	36	25	23	23
Positively charge	29	23	40	29	23	25
Theoretical PI	9.08	6.14	8.67	8.75	6.61	8.5
Aliphatic index	103.01	90.64	90.41	91.81	103.07	103.36
GRAVY	0.084	-0.177	-0.218	-0.031	0.087	0.142
Hydrogen bands	2306	2078	2522	2301	2129	2264

Thermophilic proteins are substantially more hydrophobic [85] and have a greater surface area buried upon oligomerization compared with their mesophilic homologs [86]. In contrast to Tyr and Arg, Trp is a hydrophobic amino acid with a large double-ring side chain, which generally occurs in low frequency and affects the stability of proteins (Table 6). Otherwise, it is probable that the absence of trend for Trp, is due to its low count protein stability [81]. It has been established that the aliphatic indices, which reflect the relative volume of a protein that is occupied by aliphatic side chains such as those of Val, Ile, Ala, and Leu, of proteins in thermophilic bacteria are higher than those of mesophilic proteins [80]. The aliphatic index positively affects the thermostability of globular proteins [80,87]. The aliphatic indices of proteins of thermophilic origin, particularly those with molecular weights of less than 100 kDa are considerably higher than those of mesophilic origin [80] and their increased polar surface area contributes to the greater thermal stability of the former proteins [85]. Therefore, the low molecular weight of 32 kDa of MAP30-KDEL and its high aliphatic index and very low GRAVY value, which are similar to those of the other RIPs examine, could account for its thermophilicity (Fig 6; Table 6).

### Effect of their secondary structural contents on the thermal and pH stability of RIPs

The results showed that RIPs have a high helical content (Table 7). Consistent with the results shown in Table 5, Kumar et al. 2000 previously observed that thermophilic proteins have a high helical content [82]. This feature might be explained by the high level of Arg, a helix-favoring residue and the low level of the helix-disfavoring residues His and Cys in the helices of thermophilic proteins [82]. Helixes tend to have a biased distribution of hydrophobic residues, such that they occur chiefly on one face of these structures [88]. Ala is the best helix-forming residue [89] and is therefore considered a major factor in protein thermostability (Table 5).

**Table 7:** Percentage of various secondary structures in RIPs determined using SOPMA.

RIPs	Alpha helix	Extended strand	Beta turn	Random coil
MAP30	33.22	32.17	8.04	26.57
Ricin A chain	34.09	23.22	8.61	34.08
GAP31	30.70	33.54	10.44	25.32
Curcin 1	43.34	18.09	8.19	30.38
Trichosanthin	37.03	27.78	6.67	28.52
Karasurin	34.94	27.34	7.27	30.45

Including certain residues and omitting others is a dual strategy for enhancing the stability of thermophilic proteins [82]. Regarding the residues involved in α-helical conformations, a higher content of Arg increases the number of salt bridges and stabilizes α-helices [82–83]. It is desirable to avoid having Pro and His in α-helices and to avoid the thermolabile residue Cys (Table 5). Pro occurs at a frequency of 0.7% in the α-helices of thermophilic proteins and at a frequency of 1.3% in the α-helices of mesophilic proteins [82]. Placing Pro within the interior of α-helices should be avoided because this may cause bending [90]. Secondary structural analysis revealed that MAP30 and other well-known RIPs have a rather high content of random coils (Table 7). In a random coil, the only fixed relationship between the amino acids is that between nearby residues occurring through the peptide bond [91]. Coiled regions have a higher content of small, aromatic and charged amino acids (Tables 5 and 7), which explains the high catalytic efficacy of proteins with abundant coiled regions compared with that of their counterparts from thermophilic habitats [91].

## Conclusions

The development of drug resistance by microorganisms appears to be a continuous process that began when antibiotics were discovered, and it is time for these compounds to be replaced by various plant pharmaceutical proteins and peptides. MAP30 is derived from *M*. *charantia*, the extracts of which have been used as therapeutic agents for centuries. Producing these types of proteins in plants via “molecular farming” has significant advantages in terms of cost and safety, making them a promising platform. HRs are a valuable, efficient, simple, and low-cost platform for the production of antiviral, antitumor, and antimicrobial recombinant proteins for use as therapeutic agents. In addition, foreign proteins can be recovered from HRs grown in an inexpensive and well-defined medium using simple methods. The MAP30 that was successfully expressed in HRs exhibited strong antimicrobial activity against both Gram-positive and Gram-negative bacteria and against fungi. Hairy roots have been shown to be an excellent host for the large-scale expression of various types of heterologous proteins for further structural and functional analyses. Recombinant MAP30 with broad antimicrobial activity would be a promising antibiotic candidate for commercial use in many industries and could be applied as a food and cosmetics supplement and in medical therapies. However, further studies are required to determine the activities of MAP30 against other important pathogenic bacteria, viruses and fungi.

## Abbreviations

RPESs: recombinant protein expression systems
PTMs: posttranslational modifications
PBRPESs: plant based recombinant protein expression systems
HRs: hairy roots
KDEL: Lys-Asp-: Glu: -: Leu
RIP: ribosome-inactivating protein
NOS: nopaline synthase
CaMV 35S: cauliflower mosaic virus 35S
ER: endoplasmic reticulum
GRAVY: grand average of hydropathicity

## References

[1] Zasloff M. Antimicrobial peptides of multicellular organisms. Nature. 2002;415: 389–395. 11807545 10.1038/415389a

[2] Martin KW, Ernst E. Herbal medicines for treatment of bacterial infections: a review of controlled clinical trials. J Antimicrob Chemother. 2003;51: 241–246. 12562687 10.1093/jac/dkg087

[3] Ma JKC, Drake PMW, Christou P. The production of recombinant pharmaceutical proteins in plants. Nat Rev Genrt. 2003;4: 794–805. 10.1038/nrg1177 14526375

[4] Baquero F, Martı´nez JL, Canto´n R. Antibiotics and antibiotic resistance in water environments. Curr Opin Biotech. 2008;19: 260–265. 10.1016/j.copbio.2008.05.006 18534838

[5] Verraes C, Van Boxstael S, Van Meervenne E, Van Coillie E, Butaye P, Catry B, et al Antimicrobial resistance in the food chain: a review. Int J Environ Res Public Health. 2013;7: 2643–2669. 10.3390/ijerph10072643 PMC3734448 23812024

[6] Cândido Ede S, Pinto MF, Pelegrini PB, Lima TB, Silva ON, Pogue R, et al Plant storage proteins with antimicrobial activity: novel insights into plant defense mechanisms. FASEB J. 2011;10: 3290–3305. 10.1096/fj.11-184291 21746866

[7] Rothan HA, Bahrani H, Mohamed Z, Abd Rahman N, Yusof R. Fusion of protegrin-1 and plectasin to MAP30 shows significant inhibition activity against Dengue virus replication. PLoS ONE. 2014; 10.1371/journal.pone.0094561 PMC3983197 24722532

[8] Rothan HA, Ambikabothy J, Abdulrahman AY, Bahrani H, Golpich M, Amini E, et al Scalable production of recombinant membrane active peptides and its potential as a complementary adjunct to conventional chemotherapeutics. PLoS ONE. 2015; 10.1371/journal.pone.0139248 PMC4587966 26418816

[9] Gomord V, Chamberlain P, Jefferis R and Faye L. Biopharmaceutical production in plants: problems, solutions and opportunities. TRENDS Biotech. 2005;23: 559–565. 10.1016/j.tibtech.2005.09.003 16168504

[10] Schwartz JR. Advances in *E*. *coli* production of therapeutic proteins. Curr Opin Biotech. 2001;12: 195–201. 11287237 10.1016/s0958-1669(00)00199-3

[11] Valkova R, Apostolova E, Samir N. Plant molecular farming: opportunities and challenges. J Serb Chem Soc. 2013;78: 407–415.

[12] Kuo YC, Tan CC, Ku JT, Hsu WC, Su SC, Lu CA, et al Improving pharmaceutical protein production in *O*. *sativa*. Int J Mol Sci. 2013;14: 8719–8739. 10.3390/ijms14058719 23615467 PMC3676753

[13] Gecchele E, Merlin M, Brozzetti A, Falorni A, Pezzotti M, Avesani L. A comparative analysis of recombinant protein expression in different biofactories: bacteria, insect cells and plant systems. J Vis Exp. 2015; 10.3791/52459 PMC4401374 25867956

[14] Gomord V, Faye L. Posttranslational modification of therapeutic proteins in plants Curr Opin Plant Biol. 2004;7: 2171–181. 10.1016/j.pbi.2004.01.015 15003218

[15] Grangeasse C, Stülke J, Mijakovic I. Regulatory potential of post-translational modifications in bacteria. Front Microbiol. 2015; 10.3389/fmicb.2015.00500 PMC4446998 26074895

[16] Lai JR, Huck BR, Weisblum B, Gellman SH. Design of non-cysteine containing antimicrobial b-hairpins: Structure-activity relationship studies with linear protegrin-1 analogues. Biochem. 2002;41: 12835–12842. 12379126 10.1021/bi026127d

[17] Fischer R, Emans N. Molecular farming of pharmaceutical proteins. Transgenic Res. 2000;9: 279–299. 11131007 10.1023/a:1008975123362

[18] Xu J, Ge X, Dolan MC. Towards high-yield production of pharmaceutical proteins with plant cell suspension cultures. Biotech Adv. 2011;29: 278–299. 10.1016/j.biotechadv.2011.01.002 21236330

[19] Xu J, Dolan M, Medrano G, Cramer C, Weathers. Green factory: Plants as bioproduction platforms for recombinant proteins. Biotech Adv. 2012;30: 1171–1184. 10.1016/j.biotechadv.2011.08.020 21924345

[20] Peterson RKD, Charles AJ. On risk and plant-based biopharmaceuticals. Trends Biotech. 2004; 10.1016/j.tibtech.2003.11.007 14757039

[21] Gaume A, Komarnytsky S, Borisjuk N, Raskin I. Rhizosecretion of recombinant proteins from plant hairy roots. Plant Cell Rep 2003;21:1188–93. 12819926 10.1007/s00299-003-0660-3

[22] Huet Y, Ekouna JP, Caron A, Mezreb K, Boitel-Conti M, Guerineau F. Production and secretion of a heterologous protein by turnip hairy roots with superiority over tobacco hairy roots. Biotechnol Lett. 2014;3: 181–90. 10.1007/s10529-013-1335-y 24078130

[23] Aleinein RA, Schäfer H, Wink M. Rhizosecretion of the recombinant antimicrobial peptide ranalexin from transgenic tobacco hairy roots. RRJBS Phytopathol Gene Diseas. 2015;S1: 45–55.

[24] Sharifi S, Sattari NT, Zebarjadi A, Majd A, Ghasempour H. The influence of *A*. *rhizogenes* on induction of hairy roots and β-carboline alkaloids production in *Tribulus terrestris* L. Physiol Mol Biol Plants. 2014;20: 69–80. 10.1007/s12298-013-0208-0 24554840 PMC3925472

[25] Carlín PA, Tafoya FG. Alpuche Solís A, Pérez-Molphe-Balch E. Effects of different culture media and conditions on biomass production of hairy root cultures in six Mexican cactus species. In Vitro Cell Dev Biol Plant. 2015;51: 332–339.

[26] Fischer R, Stoger E, Schillberg S, Paul C, Twyman MR. Plant-based production of biopharmaceuticals. Curr Opin Plant Biol. 2004;7:152–158. 15003215 10.1016/j.pbi.2004.01.007

[27] Wongsamuth R, Doran PM. Production of monoclonal antibodies by tobacco hairy roots. Biotechnol Bioeng. 1997;54: 401–15. 18634133 10.1002/(SICI)1097-0290(19970605)54:5<401::AID-BIT1>3.0.CO;2-I

[28] Woods RR, Geyer BC, Mor TS. Hairy-root organ cultures for the production of human acetylcholinesterase. BMC Biotech. 2008; 10.1186/1472-6750-8-95 PMC2648960 19105816

[29] Parsons J, Wirth S, Dominguez M, Bravo-Almonacid F, Giulietti AM, Rodriguez Talou J. Production of human epidermal growth factor (hEGF) by in vitro cultures of *N*. *tabacum*: effect of tissue differentiation and sodium nitroprusside addition. Int J Biotechnol Biochem. 2010;6: 131–8.

[30] Trouillard R, Hubert-Roux M, Tognetti V, Guilhaudis L, Plasson C, Menu-Bouaouiche L et al Determination of multimodal isotopic distributions: the case of a 15n labeled protein produced into hairy roots. Anal Chem. 2015; 87: 5938–5946. 10.1021/acs.analchem.5b01558 25973921

[31] Stornaiuolo M, Lotti LV, Borgese N, Torrisi MR, Mottola G, Martire Gianluca, et al KDEL and KKXX retrieval signals appended to the same reporter protein determine different trafficking between endoplasmic reticulum, intermediate compartment, and golgi complex. Mol Biol Cell. 2003;14: 889–902. 12631711 10.1091/mbc.E02-08-0468 PMC151567

[32] Martínez C, Petruccelli S, Giulietti AM, Alvarez MA. Expression of the antibody 14D9 in *N*. *tabacum* hairy roots. Elec J Biotech. 2005; 10.2225/vol8-issue2-fulltext-10

[33] Stirpe F, Ribosome-inactivating proteins: from toxins to useful proteins. Toxicon. 2013;67: 12–16. 10.1016/j.toxicon.2013.02.005 23462379

[34] de Virgilio M, Lombardi A, Caliandro R, Fabbrini MS. Ribosome-inactivating proteins: from plant defense to tumor attack. Toxins. 2010;2: 2699–2737. 10.3390/toxins2112699 22069572 PMC3153179

[35] Wang P, Tumer NE. Virus resistance mediated by ribosome inactivating proteins. Adv Virus Res. 2000;55: 325–356. 11050946 10.1016/s0065-3527(00)55007-6

[36] Arazi T, Lee Huang P, Lin Huang P, Zhang L, Moshe SY, Gal-On A, et al Production of Antiviral and Antitumor Proteins. Biochem Biophys Res Commun. 2002;292: 441–448. 11906182 10.1006/bbrc.2002.6653

[37] Lee-Huang S, Huang PL, Nara PL, Chen HC, Kung HF, Huang P, et al MAP30: a new inhibitor of HIV-1 infection and replication. FEBS letters. 1990;272:12–18. 1699801 10.1016/0014-5793(90)80438-o

[38] Lee-Huang S, Huang PL, Chen HC, Huang PL, Bourinbaiar A, Huang HI, et al Anti-HIV and anti-tumor activities of recombinant MAP30 from bitter melon. Gene. 1995;161: 151–156. 7665070 10.1016/0378-1119(95)00186-a

[39] Fan JM, Luo J, Xu J, Zhu S, Zhang Q, Gao DF, et al Effects of recombinant MAP30 on cell proliferation and apoptosis of human colorectal carcinoma LoVo cells, Mol Biotechnol. 2008;39: 79–86. 10.1007/s12033-008-9034-y 18246454

[40] Fang EF, Zhang CZ, Wong JH, Shen JY, Li CH, Ng TB. The MAP30 protein from bitter gourd (*M*. *charantia*) seeds promotes apoptosis in liver cancer cells in vitro and in vivo. Cancer Lett. 2012;324: 66–74. 10.1016/j.canlet.2012.05.005 22579806

[41] Han XH, Xue YJ, Chen DY, Shao SH, Huang H, Li XQ, et al Cloning, expression and functional analysis of MAP30 from *M*. *charantia* reveals its induction of apoptosis of the BGC-823 cells. Afr J Biotech. 2011;10: 17925–17933.

[42] Lv Q, Yang XZ, Fu LY, Lu YT, Lu YH, Zhao J, et al Recombinant expression and purification of a MAP30-cell penetrating peptide fusion protein with higher anti-tumor bioactivity. Prot Expr Purif. 2015;111: 9–17. 10.1016/j.pep.2015.03.008 25797209

[43] Caizhen G, Yan G, Ronron C, Lirong Y, Panpan C, Yuanbiao Q, et al Zirconium phosphatidylcholine-based nanocapsules as an in vivo degradable drug delivery system of MAP30, a momordica anti-HIV protein. Int J Pharm. 2015;483: 188–199. 10.1016/j.ijpharm.2015.02.021 25681721

[44] Murashige T, Skoog F. A revised medium for rapid growth and bio assays with tobacco tissue cultures. Physiol Plant. 1962;15: 473–497.

[45] Knapp JE, Chandlee JM. RNA/DNA mini-prep from a single sample of orchid tissue. Biotechniq. 1996;21: 54–56. 10.2144/96211bm11 8816235

[46] Bradford MM. A rapid and sensitive method for the quantitation of microgram quantities of protein utilizing the principle of protein-dye binding. Anal Biochem. 1976; 72: 248–254. 942051 10.1016/0003-2697(76)90527-3

[47] Laemmli UK. Cleavage of structural proteins during the assembly of the head of bacteriophage T4. Nature. 1970;227: 680–685. 5432063 10.1038/227680a0

[48] Bonev B, Hooper J, Parisot J. Principles of assessing bacterial susceptibility to antibiotics using the agar diffusion method. JAC. 2008;6: 1295–301. 10.1093/jac/dkn090 18339637

[49] Nguyen C, Bourgaud F, Forlot P, Guckert A. Establishment of hairy root cultures of Psoralea species. Plant Cell Rep. 1992;11: 424–7. 10.1007/BF00234375 24201547

[50] Alpizar E, Dechamp F, Lapeyre-Montes C, Guilhaumon B, Bertrand C, Jourdan P, et al *A*. *rhizogenes*-transformed roots of coffee (*Coffea arabica*): conditions for long-term proliferation, and morphological and molecular characterization. Ann Bot. 2008;7: 929–940. 10.1093/aob/mcn027 PMC2710235 18316320

[51] Giri A, Narasu ML. Transgenic hairy roots: recent trends and applications. Biotech Adv. 2000;18: 1–22. 10.1016/s0734-9750(99)00016-6 14538116

[52] Medina-Bolívar F, Cramer C. Production of recombinant proteins by hairy roots cultured in plastic sleeve bioreactors. Methods Mol Biol. 2004;267:351–63. 15269436 10.1385/1-59259-774-2:351

[53] Tang J, Scarth R, Fristensky B. Effects of genomic position and copy number of Acyl-ACP thioesterase transgenes on the level of the target fatty acids in *B*. *napus* L. Mol Breed. 2003;12: 71–81.

[54] Huang PL, Sun Y, Chen HC, Kung HF, Huang PL, Lee-Huang S. Proteolytic fragments of anti-HIV and anti-tumor proteins MAP30 and GAP31 are biologically active. Biochem Biophys Res Commun. 1999;262: 615–623. 10471373 10.1006/bbrc.1999.1213

[55] Meng Y, Liu S, Li J, Zhao X. Preparation of an antitumor and antivirus agent: chemical modification of a-MMC and MAP30 from *M*. *charantia* L. with covalent conjugation of polyethylene glycol. Int J Nanomed. 2012;7: 3133–3142. 10.2147/IJN.S30631 PMC3396394 22802682

[56] Roncuzzia L, Gasperi-Campani A. DNA-nuclease activity of the single-chain ribosome-inactivating proteins dianthin 30, saporin 6 and gelonin. FEBS Lett. 1996;392: 16–20. 8769306 10.1016/0014-5793(96)00776-4

[57] Ling J, Liu WY, Wang TP. Cleavage of supercoiled double-stranded DNA by several ribosome-inactivating proteins in vitro. FEBS Lett. 1994;345: 143–146. 8200446 10.1016/0014-5793(94)00421-8

[58] Ebert RF, Spryn LA. Immunotoxin construction with a ribosome-inactivating protein from barley. Bioconjug Chem. 1990;1: 331–336. 2098110 10.1021/bc00005a006

[59] Lin Huang P, Sun Y, Chen HC, Kung HF, Lee Huang P, Lee-Huang S. Proteolytic fragments of anti-HIV and anti-tumor proteins MAP30 and GAP31 are biologically active. Biochem Biophy Res Commun. 1999;262: 615–623. 10.1006/bbrc.1999.1213 10471373

[60] Vivanco JM, Savary BJ, Flores HE. Characterization of two novel type I ribosome-inactivating proteins from the storage roots of the Andean crop *M*. *expansa*. Plant Physiol. 1999;119: 1447–1456. 10198104 10.1104/pp.119.4.1447 PMC32030

[61] Ron M, Kajala K, Pauluzzi G, Wang D, A. Reynoso M, Zumstein K, et al Brady hairy root transformation using *A*. *rhizogenes* as a tool for exploring cell type-specific gene expression and function using tomato as a model. Plant Physiol. 2014;166: 455–469. 10.1104/pp.114.239392 24868032 PMC4213079

[62] Yadava A, Ockenhouse CF. Effect of codon optimization on expression levels of a functionally folded malaria vaccine candidate in prokaryotic and eukaryotic expression systems. Infect Immun. 2003;71: 4961–4969. 12933838 10.1128/IAI.71.9.4961-4969.2003 PMC187353

[63] Norkiene M, Gedvilaite A. Influence of codon bias on heterologous production of human papillomavirus type 16 major structural protein L1 in yeast. Sci World J. 2012; 10.1100/2012/979218 PMC3356764 22645496

[64] Kanaya S, Yamada Y, Kudo Y, Ikemura T. Bacillus subtilis tRNAs: gene expression level and species-specific diversity of codon usage based on multivariate analysis. Gene. 1999;238: 143–155. 10570992 10.1016/s0378-1119(99)00225-5

[65] Murray C, Sutherland PW, Phung MM, Lester MT, Marshall RK, Christeller JT. Expression of biotin-binding protein, avidin and streptavidin, in plant tissues using plant vacuolar targeting sequences. Transgenic Res. 2002;11: 199–214. 12054353 10.1023/a:1015237610263

[66] Frigerio L, Jolliffe NA, Di Cola A, Felipe DH, Paris N, Neuhaus JM et al The internal propeptide of the ricin precursor carries a sequence specific determinant for vacuolar sorting. Plant Physiol. 2001;126: 167–175. 11351080 10.1104/pp.126.1.167 PMC102291

[67] Wang F, Chi C, Wang L, Qiao Y, Jin X, Ding G. Gene cloning and expression of MAP30 in *P*. *pastoris*. Biotech Biotechnol Equip. 2014;28: 136–139. 10.1080/13102818.2014.901667 PMC4433928 26019499

[68] Irvin JD. Antiviral proteins from Phytolacca In: Chessin M, DeBorde D, Zipf A, editors. Antiviral proteins in higher plants. CRC Press: Boca Raton; 1995 pp. 65–94.

[69] Nicolas E, Goodyer ID, Taraschi TF. An additional mechanism of ribosome-inactivating protein cytotoxicity: degradation of extrachromosomal DNA. Biochem J. 1997;327: 413–417. 9359409 10.1042/bj3270413 PMC1218809

[70] Boundless. Boundless chemistry. In: Kindle editor. The effect of pH on solubility. 2013. pp. 490–6881.

[71] Ruckenstein E, Shulgin IL. Effect of salts and organic additives on the solubility of proteins in aqueous solutions. Adv Colloid Interface Sci. 2006;16: 123–126. 10.1016/j.cis.2006.05.018 16814736

[72] Zeng M, Zheng M, Lu D, Wang J, Jiang W, Sha O. Anti-tumor activities and apoptotic mechanism of ribosome-inactivating proteins. Chin J Cancer. 2015; 10.1186/s40880-015-0030-x PMC4593346 26184404

[73] Upadhyay A, Upadhyaya I, Kollanoor-Johny A, Venkitanarayanan K. Combating pathogenic microorganisms using plant-derived antimicrobials: a minireview of the mechanistic basis. BioMed Res Inter. 2014; 10.1155/2014/761741 PMC4178913 25298964

[74] Wang YX, Jacob J, Wingfield PT, Palmer I, Stahl SJ, Kaufman JD et al Anti-HIV and anti-tumor protein MAP30, a 30 kDa single-strand type-I RIP, shares similar secondary structure and b-sheet topology with the A chain of ricin, a type-II RIP. Protein Sci. 1999a; 9:138–144. 10.1110/ps.9.1.138 PMC2144446 10739256

[75] De Bolle MFC, Osborn RW, Goderis IJ et al Antimicrobial peptides from *Mirabilis jalapa* and *Amaranthus caudatus*: expression, processing, localization and biological activity in transgenic tobacco. Plant Mol Biol. 1996;31: 993–1008. 8843942 10.1007/BF00040718

[76] Zhang Y, Lewis K. Fabatins: new antimicrobial plant peptides. FEMS Microbiol Lett. 1997;149: 59–64. 9103978 10.1111/j.1574-6968.1997.tb10308.x

[77] Sharon N, Ofek I. Mannose specific bacterial surface lectins In: Mirelman D editor. Microbial lectins and agglutinins. John Wiley and Sons Inc: New York; 1986 pp. 55–82.

[78] Roberts WK, Selitrennikoff CP. Isolation and characterization of two antifungal proteins from barley. Biochim Biophys Acta. 1986;880: 161–170. 3942788 10.1016/0304-4165(86)90076-0

[79] Remi SNR, McDonald KA, Jackman AP, Girbes T, Iglesias R. Bifunctional plant defense enzymes with chitinase and ribosome inactivating activities from *T*. *kirilowii* cell cultures. Plant Sci. 1997;130: 145–150.

[80] Kombrick E, Schroeder M, Hahlbrock K. Several pathogenesis-related proteins in potato are 1,3b-glucanases and chitinases. Proc Natl Acad Sci USA. 1988;85: 782–786. 16578829 10.1073/pnas.85.3.782 PMC279639

[81] Ikai AJ. Thermostability and aliphatic index of globular proteins. J Biochem. 1980; 88: 1895–1898. 7462208

[82] Russell RJM, Ferguson JMC, Haugh DW, Danson MJ, Taylor GL. The crystal structure of citrate synthase from the hyperthermophilic archaeon *Pyrococcus furiosus* at 1.9 A resolution. Biochem. 1997;36: 9983–9994. 9254593 10.1021/bi9705321

[83] Kumar S, Tsai Chung-Jung, Nussinov Ruth. Factors enhancing protein thermostability. PDEC. 2000;13: 179–191. 10775659 10.1093/protein/13.3.179

[84] Jeffrey GA, Saenger W. Hydrogen bonding in biological structures 1st ed. Berlin: Springer-Verlag press; 1991.

[85] Bogan AA, Thorn KS. Anatomy of hot spots in protein interfaces. J Mol Biol. 1998; 280: 1–9. 9653027 10.1006/jmbi.1998.1843

[86] Haney P, Konisky J, Koretke KK, Luthey-Schulten Z, Wolynes PG. Structural basis for thermostability and identification of potential active site residues for adenylate kinases from the archaeal genus methanococcus. Proteins. 1997;28: 117–130. 9144797 10.1002/(sici)1097-0134(199705)28:1<117::aid-prot12>3.0.co;2-m

[87] Salminen T, Teplyakov A, Kankare J, Cooperman BS, Lahti R, Goldman A. An unusual route to thermostability disclosed by the comparison of *Thermus thermophilus* and *E*. *coli* inorganic pyrophosphatases. Protein Sci. 1996;5: 1014–1025. 8762133 10.1002/pro.5560050604 PMC2143442

[88] Saha A, Jibran AMD, Muhammad AKU, Ma A. Computational analysis of bovine alpha-1 collagen sequences. Bioanformation. 2013; 9: 42–48. 10.6026/97320630009042 PMC3563415 23390343

[89] Stapley BJ, Creamer TP. A survey of left-handed polyproline II helices. Protein Sci. 1999;8: 587–95. 10091661 10.1110/ps.8.3.587 PMC2144280

[90] Sriprapundh D, Vieille C, Zeikus JG. Molecular determinants of xylose isomerase thermal stability and activity: analysis of thermozymes by site-directed mutagenesis. Protein Eng. 2000;13: 259–65. 10810157 10.1093/protein/13.4.259

[91] Kumar S, Bansal M. Structural and sequence characteristics of long alpha helices in globular proteins. Biophys J. 1996;71: 1574–1586. 8874031 10.1016/S0006-3495(96)79360-8 PMC1233624

